# Effects of Anaerobic Fermentation on Black Garlic Extract by *Lactobacillus*: Changes in Flavor and Functional Components

**DOI:** 10.3389/fnut.2021.645416

**Published:** 2021-05-21

**Authors:** Li Ma, Chengying Zhao, Jifeng Chen, Jinkai Zheng

**Affiliations:** ^1^School of Life Sciences, Zhengzhou University, Zhengzhou, China; ^2^Institute of Food Science and Technology, Chinese Academy of Agricultural Sciences, Beijing, China

**Keywords:** black garlic extract, fermentation, *Lactobacillus*, sensory, functional components

## Abstract

The purpose of this study is to investigate the potential application of probiotics in the development of novel functional foods based on black garlic. The single-factor analysis (extraction temperatures, solid-to-liquid ratios, and extraction times) and the response surface methodology were firstly used to optimize hot water extraction of soluble solids from black garlic. The optimal extraction conditions were temperature 99.96°C, solid-to-liquid ratio 1:4.38 g/ml, and extracting 2.72 h. The effects of *Lactobacillus* (*Lactobacillus plantarum, Lactobacillus rhamnosus*, and co-culture of them) fermentation on the physicochemical properties of black garlic extract broth were studied for the first time. Artificial and electronic sensory evaluations demonstrated that fermentation significantly influenced the sensory characteristics. The variations of metabolites in different broth samples (S1, unfermented; S2, 1-day fermentation by *L. plantarum*; S3, 2-day fermentation by *L. rhamnosus*; and S4, 1-day fermentation by co-cultured *Lactobacillus*) were further investigated by gas chromatography-mass spectrometry and liquid chromatography-mass spectrometry/mass spectrometry analysis. As a result, *Lactobacillus* fermentation significantly reduced the pH; increased the contents of the total acid, amino nitrogen, total polyphenol, and total flavonoid; and reduced the content of 5-hydroxymethylfurfural (a carcinogenic component) by 25.10–40.81% in the black garlic extract. The contents of several components with unpleasant baking flavors (e.g., furfural, 2-acetylfuran, and 5-methyl furfural) were reduced, whereas the contents of components with green grass, floral, and fruit aromas were increased. More importantly, the contents of several functional components including lactic acid, Gly-Pro-Glu, sorbose, and α-CEHC (3,4-dihydro-6-hydroxy-2,5,7,8-tetramethyl-2H-1-benzopyran-2-propanoic acid) were increased after *Lactobacillus* fermentation. The results demonstrated the potential of probiotic fermentation to improve the quality of black garlic. This work will provide an insight into the strategic design of novel black garlic products and facilitate the application of black garlic in functional foods.

## Introduction

Black garlic (*Allium sativum* L.) is an emerging processed food obtained by fresh garlic under high temperature (60–90°C) and high humidity (60–80%) for 60–90 days (1, 2). In recent years, black garlic has been highly relished, especially by consumers in Asian countries such as Japan, Singapore, and China (2, 3). Unlike fresh garlic, the pungent tasting allicin is easily decomposed and converted into antioxidants during the production of black garlic (3, 4). At the same time, the depolymerization of garlic polysaccharide significantly increases the reducing sugar content, and organic acids are produced by a series of reactions that endow black garlic with a sweet and sour taste (1). In addition, cell wall polysaccharides degrade under high-temperature conditions, resulting in tissue softening and a chewy, jelly-like texture (1, 3). Moreover, evidence suggests that black garlic has many remarkable nutritional and health benefits, including anti-oxidative, anti-allergic, anti-diabetic, anti-inflammatory, and anti-cancer effects (5–7). Therefore, research on black garlic has attracted extensive attention, especially on its development and application in functional foods.

An investigation on the components of black garlic is of particular importance, as they determine its physicochemical and sensory properties. The functional components of black garlic are composed mainly of sulfur compounds, carbohydrates, amino acids, melanoidin (MLD), polyphenols, and 5-hydroxymethylfurfural (5-HMF) (7). Sulfides endow black garlic with its unique flavor and play an important role in its anti-cancer bioactivity (8, 9). At high temperatures, carbohydrates and amino acids can be transferred to antioxidant compounds (e.g., MLD) by a series of Maillard reactions of non-enzymatic browning (10–12). MLD plays a key role in the changes in physical, chemical, and sensory properties as well as the biological effects of food, including antioxidant, antitumor, antibacterial, anti-inflammatory, hypoglycemic, and antihypertensive activities (11, 13, 14). In addition, the content of polyphenols, flavonoids, and phenolic acids in black garlic can be increased by 7–11-, 1–5-, and 4–8-fold in comparison with fresh garlic, respectively, which could significantly increase its antioxidant effects (9). A higher temperature has been used to shorten the processing time of black garlic, which results inserious declines in the taste, flavor, and health value of black garlic in the market and even serious accumulation of harmful substances (e.g., 5-HMF).

Probiotics, defined as living microorganisms that can bring health benefits to the host when used in an appropriate amount, have been used to produce fruit and vegetable juice products in the market with a pleasant taste and flavor, as well as functional foods (15, 16). Probiotic fermentation is widely used in the food industry. For example, *Lactobacillus plantarum*-fermented garlic can increase the content of diallyl trisulfide to retain H_2_S-releasing activity, which can regulate cardiovascular functions and anti-cancer activities (17). In RAW 264.7 cells, a blend of *Leuconostoc mesenteroides*-fermented garlic and *Cirsium setidens* Nakai can promote antioxidant and immune activities (18). In animal studies, *L. plantarum* BL2-fermented garlic extract promotes weight loss in diet-induced obese mice (19); *Saccharomyces cerevisiae* (KCTC7910)-fermented black garlic can increase antioxidant activity, protect the liver and kidneys, lower blood lipid levels, and promote weight loss (20). Currently, there are few reports on the fermentation and extraction of black garlic using the *L. plantarum-* and *Lactobacillus rhamnosus*-fermented extracts on the market. Black garlic contains a high level of reducing sugar, which can provide energy for probiotic metabolism. Conversely, the fermentation process may also affect the flavor and functional components of black garlic.

Herein, we aimed to investigate the effects of *Lactobacillus* (*L. plantarum* and *L. rhamnosus*) fermentation on black garlic extract, especially the changes in flavor and functional components. The conditions of soluble solid extraction from black garlic were optimized by a single-factor analysis and the response surface methodology. The variations in physicochemical properties as well as in artificial and electronic sensory characteristics were analyzed systematically. To reveal the mechanisms of these variations, gas chromatography–mass spectrometry (GC-MS) and liquid chromatography–mass spectrometry (LC-MS) were used to investigate the effects of probiotic fermentation on the components of black garlic, especially flavor and functional components.

## Materials and Methods

### Materials and Reagents

*L. plantarum* (BNCC336421) and *L. rhamnosus* (BNCC185356) were purchased from Beijing Be Na Culture Collection Technology Co. (Beijing, China). Processed black garlic was provided by Heze Tianhong Fruits and Vegetables Co. (Shandong, China).

### Optimization of Black Garlic Extraction Conditions

Single-factor and response surface optimization experiments were used to optimize the hot water extraction of soluble solids from black garlic (21). In the single-factor experiments, samples (50 g) of peeled and cleaned black garlic were soaked in ultrapure water for extraction at various temperatures (60, 70, 80, 90, and 100°C), solid-to-liquid ratios (1:4, 1:5, 1:6, 1:7, and 1:8 g/ml), and extraction times (1.0, 1.5, 2.0, 2.5, and 3.0 h). The black garlic mixture was vacuum-filtered through a 300-mesh nylon cloth to obtain the black garlic extraction solution. The soluble solid content was measured using a portable Abbe refractometer (WAY-2S, Yice, Shanghai, China).

After preliminary screening, the optimal extraction conditions were determined, then a three-factor, three-level Box–Behnken design was performed to optimize the extraction conditions. The extraction temperature, solid-to-liquid ratio, and extraction time were selected as the three main variables, and the soluble solid content of the extract was defined as the response of the combined independent variables. The soluble solid content (*Y*) was calculated according to Equation 1 (22):


$$\begin{array}{l} Y=\beta_{0}+\overset{n}{\underset{i=1}{\sum }}\beta_{i}X_{i}+\overset{n}{\underset{i=1}{\sum }}\beta_{ii}{X_{i}}^{2}+\overset{n}{\underset{j=i+1}{\sum }}\beta_{ij}X_{j} \end{array}$$

where *Y* is the predicted response (soluble solid content); β_0_ is a constant; β_*i*_, β_*ii*_, and β_*ij*_ represent the coefficients of the linear, quadratic, and interaction effects, respectively; and *X*_*i*_ and *X*_*j*_ are the independent variables. The fit of the model was evaluated by coefficients of determination (*R*^2^), *P*-values, lack-of-fit test, and root mean square errors. The validation of the model was performed by applying the optimized extraction conditions of the independent variables and comparing them with the predicted values.

### Fermentation of Black Garlic Extract by *Lactobacillus*

Under anaerobic conditions (95% N_2_ and 5% CO_2_), 1 g freeze-dried powder of *L. plantarum* or *L. rhamnosus* was inoculated on de man rogosa and sharpe (MRS) agar medium and cultured for 48 h. Vigorous colonies were activated twice in succession, inoculated with vigorous growth in ring 2 into 100 ml MRS broth, and cultured at 0.5% dissolved oxygen and 37°C for 24 h. The above processes were all carried out in an anaerobic incubator (YQX-1, Yuejin, Shanghai, China). Then, 10 ml of rejuvenation culture solution was centrifuged at 5,000*g* for 10 min at 4°C, and the precipitate was used for the fermentation of black garlic extract broth.

Black garlic extract broth was pasteurized at 100°C for 10 min. Under anaerobic conditions, rejuvenated probiotics were diluted to 10^10^ colony-forming units (CFUs)/ml. The sterilized black garlic extract broth was fermented with 1% *L. plantarum* dilution, *L. rhamnosus* dilution, or a 1:1 mixture of the two for 0–4 days. Subsequently, the fermented black garlic extract broths were stored at −80°C until used.

### Measurement of pH and Viable Bacterial Counts in Black Garlic Fermentation Broth

#### pH

At room temperature (25°C), a pH meter was used to measure the pH of the black garlic fermentation broth.

#### Viable Bacterial Counts

The viable bacterial counts were determined according to previous studies with minor modifications (23). After *Lactobacillus* was fermented for 1 day, 10 ml of fermentation broth was centrifuged at 5,000 *g* for 10 min at 4°C. The supernatant was discarded, and 10 ml sterilized PBS (pH 7.2) was added, which was diluted in a 10-fold series. Then, 50 μl of a different dilution was coated on MRS agar medium and cultured upside down at 37°C for 24 h, respectively. When the number of CFUs on the culture medium counted to 30–100, it was used to calculate the number of viable bacteria in the fermentation broth.

### Measurement of Total Acid and Amino Nitrogen Contents of Black Garlic Fermentation Broth

Black garlic fermentation broth (20.00 ml, 1:10 dilution) was transferred to a beaker and stirred. The solution was titrated with 0.05 mol/l NaOH solution to pH 8.20, and the consumed volume of NaOH solution was recorded. Then, 2.50 ml formaldehyde was added to the mixture, which was titrated with 0.05 mol/l NaOH to pH 9.20, and the amount of NaOH solution consumed was recorded. Distilled water (20.00 ml) was used as the blank group. The consumed volumes of NaOH solution were used to calculate the total acid content and the amino nitrogen (amino-N) content (20).

### Measurement of the Total Polyphenol Content of Black Garlic Fermentation Broth

The total polyphenol content was determined according to previous studies with minor modifications (18, 24). We added 1.25 ml 10% Folin–Ciocalteu reagent (*v*/*v*) and 1 ml 7.50% sodium carbonate solution (*w*/*v*) to 0.50 ml black garlic fermentation broth (1:50 dilution). The mixture was incubated in a 45°C water bath for 40 min. The absorbance was measured at 765 nm, and the total polyphenol content was measured using gallic acid equivalents as the calibration curve standard.

### Measurement of Total Flavonoid Content of Black Garlic Fermentation Broth

The total flavonoid content was determined according to a previous report with minor modifications (18). First, 5.00 ml black garlic fermentation broth (1:20 dilution) was added to 0.30 ml 5% NaNO_2_ solution (*w*/*v*) and incubated for 5 min. Then, 0.30 ml 10% aluminum chloride solution (*m*/*v*) was added, followed by incubation for 6 min. Next, 2.00 ml NaOH solution (1 M) was added, and the mixture was adjusted to a volume of 10 ml by addition of distilled water and incubated for 15 min. The absorbance at 510 nm was measured using a microplate reader. The absorbance of quercetin equivalents was used as the standard curve to calculate the total flavonoid content.

### Measurement of the Reducing Sugar Content of Black Garlic Fermentation Broth

Determination of the reducing sugar content was performed using the DNS method (25). First, 750 μl DNS reagent was added to 1.00 ml black garlic fermentation broth (1:400 dilution) and mixed evenly by vortexing. Then, 100.00 μl 10% sodium hydroxide solution (*w*/*v*) was added to the mixture and mixed well. The samples were incubated at 100°C for 15 min and then cooled rapidly, and the absorbance was measured at 540 nm using a microplate reader. The absorbance of glucose sugar was measured as the standard curve to calculate the reducing sugar content.

### Measurement of the 5-HMF Content of Black Garlic Fermentation Broth by High-Performance Liquid Chromatography

The content of 5-HMF in black garlic fermentation broth was determined according to previous studies with slight modification (26). The black garlic fermentation broth was diluted (1:100) and filtered through a 0.22-μm aqueous phase filter membrane. The 5-HMF content was determined by a high-performance liquid chromatography (HPLC) device equipped with a photodiode array detector (L2455; Hitachi, Tokyo, Japan). The detection wavelength was 284 nm, and the column was a ZORBAX Eclipse Plus-C18 (50 × 2.1 mm, 1.8 μm; Agilent Technologies, Santa Clara, CA, USA). The mobile phase was distilled water and acetonitrile (88:12, *v*/*v*), the flow rate was 1.0 ml/min, the column temperature was 25°C, and the injection volume was 20 μl.

### Sensory Evaluation of Black Garlic Fermentation Broth

#### Artificial Sensory Evaluation

After fermentation of the black garlic broth, an artificial sensory evaluation was performed by a well-trained food evaluation team composed of 15 members in the sensory room assigned by the Institute of Agricultural Products Processing. The black garlic fermentation broth samples were numbered and randomly provided to each team member individually. The same individuals participated in all evaluations, and all were blinded to the samples tested, while water and salt-free biscuits were provided between samples for palate cleansing. Fifteen attributes related to appearance, smell, taste, and touch were scored on the unstructured evaluation form. The scoring standard was an 8-cm hedonic scale with intensity descriptors. The direction extends from the center to the outside, and the intensity increases (1: low and 8: high). The additional attributes about the overall sensory preferences of each sample were given on the same scale, defining the overall assessment (27).

#### Electronic Sensory Evaluation

The electronic eye, electronic nose, and electronic tongue were used to analyze the color, aroma, and taste of the black garlic fermentation broth using a bionic system to analyze the sensory attributes (28). Color was expressed as L^*^ (luminance), a^*^ (red-green), and b^*^ (yellow-blue). The color difference (Δ*E*) was calculated according to Equation 2:


$$\begin{array}{l} \Delta E=\sqrt{{\left(L^{*}-{L_{0}}^{*}\right)}^{2}+{\left(a^{*}-{a_{0}}^{*}\right)}^{2}+{\left(b^{*}-{b_{0}}^{*}\right)}^{2}} \end{array}$$

where L^*^, a^*^, and b^*^ represent the test group and $L_{0}^{*}$, $a_{0}^{*}$ and $b_{0}^{*}$ represent the control group. The electronic nose distinguishes the difference in sample aroma based on 10 gas sensors with different selection modes. Specifically, W1C, W5S, W3C, W6S, W5C, W1S, W1W, W2S, W2W, and W3S are sensitive to aromatic compounds, nitrogen oxides, ammonia and aromatic compounds, hydrogen, alkanes and aromatic compounds, methane, sulfur compounds, ethanol, aromatic and organic sulfur compounds, and alkanes. A principal component analysis (PCA) was used to identify the fragrance based on the sensor response value signal. The electronic tongue was based on five sensors that are sensitive to acid, sweet, bitter, salty, and fresh to identify samples, and we used PCA to identify taste based on the sensor response signal.

### Headspace Solid-Phase Microextraction–GC–MS Analysis of Black Garlic Fermentation Broth

Headspace solid-phase microextraction (HS-SPME) was used to separate and concentrate the volatile components in the black garlic fermentation broth, which were then analyzed by GC-MS (QP 2010 Plus; Shimadzu, Japan) (21, 29). The SPME procedure was as follows: 2.00 ml black garlic extract and 100 μl (0.38 μg/ml) cyclohexanone were transferred into a 20-ml headspace bottle with a 20-mm aluminum cap and a 20-mm silicon/polytetrafluoroethylene diaphragm. The sample vial was incubated at 50°C for 30 min in the dark. The SPME needle (75-μm divinylbenzene/carboxen/polydimethylsiloxane) was aged for 3 min at 250°C until no residue was observed, inserted into the headspace bottle at 50°C for 30 min, and immediately retracted and inserted into the GC inlet. The desorption temperature and time were 250°C and 5 min, respectively. The GC oven temperature was programmed as follows: 40°C for 3 min, increase to 160°C at a rate of 4°C/min, hold for 3 min, then increase to 250°C at a rate of 7°C/min, and hold for 5 min. GC conditions were as follows: carrier gas, high-purity nitrogen; split, 1:30; flow rate, 1.00 ml/min; inlet temperature, 230°C; and flame ionization detector temperature, 250°C. A DB-WAX capillary column (100 mm × 0.25 mm i.d., 0.25 μm, Agilent Technologies, Santa Clara, CA, USA) was used. The MS conditions were as follows: ionization mode, electron bombardment (electron ionization source); ion source temperature, 230°C; transmission line temperature, 250°C; electron energy, 70 eV; and scanning range, *m*/*z* 30–600. The volatile compounds of the black garlic fermentation broth were identified by comparing their retention time, CAS number, linear retention indices (Kovats indices), serial number, mass spectra, and principal fragments with those in the NIST 11 standard library. Based on the GC peak area of the internal standard (cyclohexanone), the relative contents of each volatile compound in the four samples were calculated.

### LC–Quadrupole Time-of-Flight–MS/MS Analysis of Black Garlic Fermentation Broth

LC–quadrupole time-of-flight (Q-TOF)–MS/MS (1200 LC, 6540 UHD Q-TOF; Agilent Technologies) was used to initially identify the polar metabolites in the broth. The method for LC–Q-TOF–MS/MS analysis was slightly modified according to previous reports (30). The fermentation broth was diluted (1:100) with ultrapure water, mixed with an equal volume of methanol, filtered through a 0.25-μm organic phase membrane, and stored at −20°C until used. The mobile phases were 0.10% (*v*/*v*) formic acid in ultrapure water (phase A) and 0.1% (*v*/*v*) formic acid in methanol (phase B) at a flow rate of 0.4 ml/min. The gradient was as follows: start at 5% B, increase from 5 to 10% B over 5 min, increase from 10 to 100% B over 25 min, and hold at 100% B for 5 min. The setup was as follows: chromatographic column, ZORBAX Eclipse Plus-C18 (50 × 2.1 mm i.d., 1.8 μm, Agilent Technologies); column temperature, 40°C; and injection volume, 10 μl. The parameters of the dual electrospray ionization source and mass spectrometer were as follows: capillary, skimmer, and Q1 voltages set to 3,500 V, 60 eV, and 120 V, respectively; N_2_ atomizing gas flow rate, 40 psi; drying gas flow rate, 10 ml/min; temperature, 325°C; and octopole radiofrequency voltage, 750 V. Two values of collision energy, 20 and 40 eV, were combined with MS/MS data at two fragmentation levels. The *m*/*z* range of 30–1,700 was used for MS and MS/MS; data were collected at a rate of one spectrum per second in the extended dynamic range mode. The auto MS/MS mode was configured with two maximum precursors per cycle and an exclusion window of 1 min after two consecutive selections of the same precursor. To ensure the expected mass accuracy of the recorded ions, *m*/*z* 121.0509 (protonated purine), *m*/*z* 922.0098 [protonic hexakis (1H, 1H, 3H-tetrafluoropropoxy) phosphazene (HP) at *m*/*z* 921], and the signals at *m*/*z* 112.9856 (trifluoroacetic acid anion) and *m*/*z* 1,033.9881 (HP at *m*/*z* 921) in a negative ionization mode were continuously calibrated internally.

The MassHunter workstation software (version B 08.00 qualitative analysis; Agilent Technologies) was used to extract potential molecular features (MFs) from all datasets. All ions with a single charge count exceeding 1,500 were taken into account in the extraction algorithm. The isotope distribution of MF inclusions should be defined by at least two ions (peak spacing *m*/*z* tolerance = 0.0025 and mass accuracy = 10.0 ppm). In addition to [M + H]^+^ and [M – H]^−^ ions, there were also adducts in positive ionization mode (+Na, +K, and +NH_4_), negative ionization mode (+Cl and +HCOO), and neutral loss caused by dehydration to identify MFs corresponding to the same potential metabolite. The raw data were filtered with a minimum count level of 3,000 for samples analyzed in positive ionization mode and 2,500 in negative ionization mode. The resulting MFs were tentatively identified by searching MS and MS/MS information in the METLIN Metabolite and Chemical Entity Database (http://metlin.scripps.edu/), and an accuracy error limit of 5 ppm was set for identification.

### Statistical Analysis

All experiments were conducted in triplicate, and the data were expressed as mean ± standard deviation (SD). The statistical methods of this study mainly used the analysis of variance (ANOVA) and a *post-hoc* least significant difference (LSD) test to calculate the difference between the treatments. The difference was significant at *P* < 0.05. IBM Statistical SPSS 25 (IBM Corporation, Chicago, IL, USA) was used for the statistical analysis of data. Design Expert 10 (Stat-Ease, Minneapolis, MN, USA) was used for the analysis in response surface optimization experiment. Origin 2019 software (OriginLab Corporation, Hampton, MA, USA) was used to draw graphics.

## Results and Discussion

### Effects of Extraction Conditions on Soluble Solids and Response Surface Optimization

**Figure 1:**
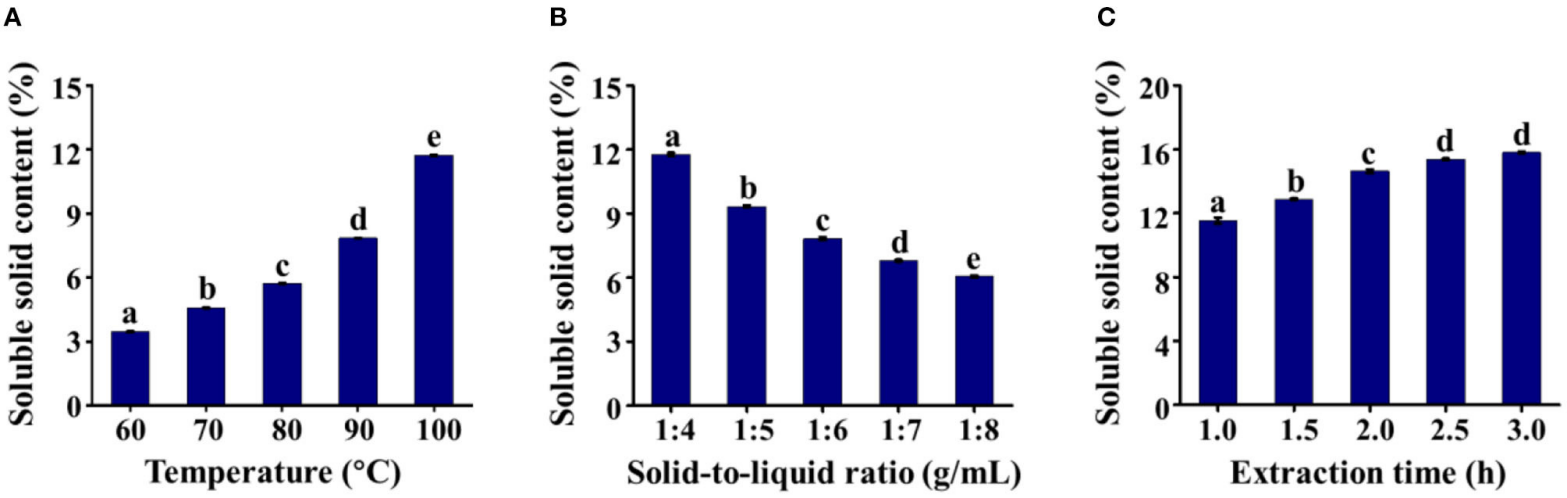
The soluble solid contents of black garlic extracts under different **(A)** extraction temperatures, **(B)** solid-to-liquid ratios, and **(C)** extraction times. Data are means ± SD (*n* = 3).

Hot water extraction is economical, convenient, and simple and is commonly used for dietary preparation in daily life. Here, we firstly assessed the effects of the extraction temperature, solid-to-liquid ratio, and extraction time on the soluble solid contents of black garlic using single-factor experiments. As shown in Figure 1A, as the extraction temperature increased (especially above 80°C), the content of soluble solids increased significantly (*P* < 0.05), which might be due the destruction of plant cell walls by high temperatures (31). The effect of the solid-to-liquid ratio was subsequently investigated at 100°C (Figure 1B). When the soluble solids in the solvent extraction were not saturated, the concentration of soluble solids decreased as the solid-to-liquid ratio increased (31). With prolongation of the extraction time, the soluble solid content increased from 1 to 2.5 h and then remained constant (Figure 1C). As a result, the parameters selected for the following studies were as follows: extraction temperature of 100°C, solid-to-liquid ratio of 1:4, and extraction time of 2.5 h.

To further increase the extraction rate of soluble solids, the response surface method was used to optimize the extraction conditions based on the above results from single-factor experiments. The experiments were performed based on a three-level Box–Behnken design (Table 1). The obtained regression model of the relationship between soluble solids in black garlic extract and extraction conditions was fitted using a second-order equation:


$$\begin{array}{l} y=+22.36250-0.53938\times A-0.38125\times B-0.62500\\ \;\times C+0.005438\times A^{2}+0.21875\times B^{2}+0.05000\\ \;\times C^{2}-0.036250\times \mathrm{AB}+0.03375\times \mathrm{AC}-0.21250\times \;\text{BC} \end{array}$$

where *y* was the content of soluble solids in the black garlic extract, and *A, B*, and *C* are the extraction temperature, solid-to-liquid ratio, and extraction time, respectively. The analysis results of the model were significant (*P* < 0.05), with an *F*-value of 114.384, demonstrating that the measured and predicted values of the model had high consistency. The coefficient of determination (*R*^2^ = 0.9932) indicated the high degree of fit. Only 0.0068% of the variation could not be explained by the model. The adjusted coefficient of determination (0.9846) was close to the coefficient of determination, and the comprehensive evaluation model had good statistical significance.

Table 1Box–Behnken experimental design and the effects of time, temperature, and solid-to-liquid ratio on hot water extraction of soluble solids from black garlic.

**Table:** 

**No**.	***A* temperature (^**°**^C)**	***B* solid/liquid ratio (g/ml)**	***C* time (h)**	***Y* content of soluble solids (%)**
1	80	4	3	8.4
2	80	4	1	5.7
3	80	6	2	4.0
4	100	6	3	11.2
5	100	8	2	7.4
6	80	6	2	4.0
7	60	4	2	3.8
8	100	4	2	14.7
9	60	6	1	2.6
10	60	8	2	2.3
11	80	6	2	4.0
12	60	6	3	3.4
13	100	6	1	7.7
14	80	8	3	3.3
15	80	6	2	4.0
16	80	6	2	4.0
17	80	8	1	2.3

**Table:** 

**Source**	**Sum of squares**	**Degree of freedom**	**Mean square**	***F*** **-value**	***P*** **-value**
Model	185.02	9	20.56	114.44	<0.0001
*A*	104.40	1	104.40	581.16	<0.0001
*B*	37.41	1	37.41	208.25	<0.0001
*C*	8.00	1	8.00	44.53	0.0003
*AB*	8.41	1	8.41	46.82	0.0002
*AC*	1.82	1	1.82	10.15	0.0154
*BC*	0.72	1	0.72	4.02	0.0849
*A* ^2^	19.92	1	19.92	110.88	<0.0001
*B* ^2^	3.22	1	3.22	17.94	0.0039
*C* ^2^	0.01	1	0.011	0.06	0.8157
Residual	1.26	7	0.18		
Lack of fit	1.26	3	0.42		
*R* ^2^	0.9932				
Adj *R*^2^	0.9846				
Regression equation	*y* = +22.36250–0.53938 × *A*−0.38125 × *B*−0.62500 × *C* + 0.005438 × *A*^2^ + 0.21875 × *B*^2^ + 0.05000 × *C*^2^-0.036250 × *AB* + 0.03375 × *AC*−0.21250 × *BC*				

**Figure 2:**
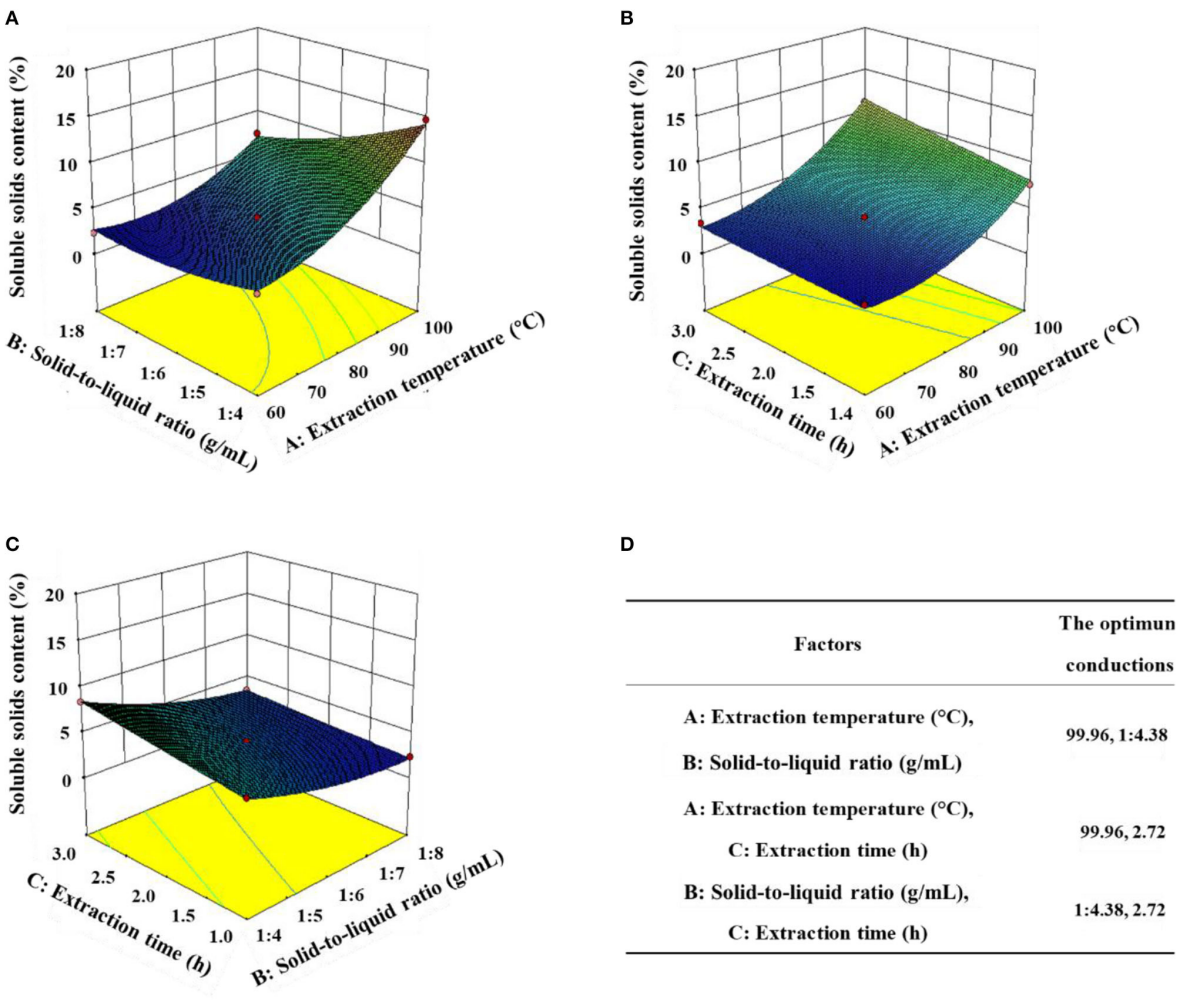
Three-dimensional response surface methodology to evaluate the effects of the **(A)** solid-to-liquid ratio/extraction temperature, **(B)** extraction time/extraction temperature, and **(C)** extraction time/solid-to-liquid ratio on the soluble solid content of black garlic extract. **(D)** The optimal extraction conditions determined by the response surface methodology. Data are means ± SD (*n* = 3).

A comprehensive evaluation of the effects of each independent extraction variable and their interactions on soluble solids in black garlic extract was conducted using a three-dimensional response surface diagram to obtain the best extraction conditions (Figure 2). In Figure 2A, when the extraction time was 1 h, the high temperature and low solid-to-liquid ratio increased the soluble solid content, and it was the highest at 99.96°C with a solid-to-liquid ratio of 1:4.4 (Figure 2D). When the solid-to-liquid ratio was 1:4 g/ml, long-term and high-temperature extraction was better than short-term and low-temperature extraction. However, with further extension of the extraction time, the soluble solid content no longer significantly increased (Figure 2B). When the extraction temperature was fixed at 100°C, the soluble solids first increased as the extraction time increased and then decreased at a high solid-to-liquid ratio. The highest soluble solid content was obtained at an extraction time of ~3 h and a solid-to-liquid ratio of ~1:4 g/ml (Figure 2C). According to the prediction of the corresponding surface, the best black garlic extraction conditions were an extraction temperature of 99.96°C, a solid-to-liquid ratio of 1:4.38 g/ml, and extraction of 2.72 h. A verification experiment was performed under the optimal conditions, and the comprehensive evaluation value was 19.04%, which was close to the predicted value of 19.28%, indicating that the model optimization of the black garlic extraction process was reliable (21).

### Effects of Fermentation on the Physicochemical Properties of Black Garlic Broth

**Figure 3:**
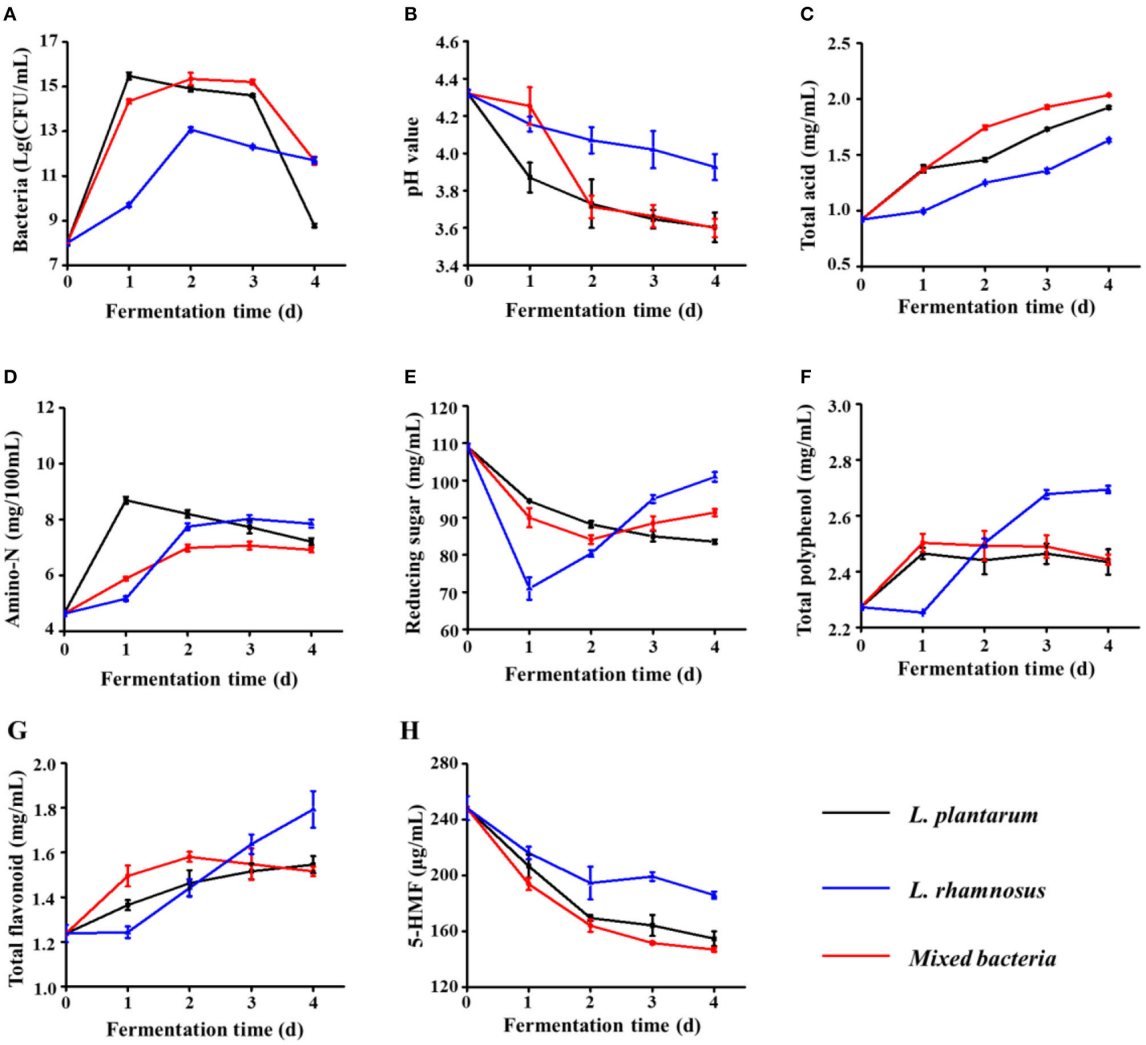
The **(A)** viable bacterial counts, **(B)** pH, **(C)** total acid contents, **(D)** amino nitrogen (amino-N) contents, **(E)** total polyphenol contents, **(F)** total flavonoid contents, **(G)** reducing sugar contents, and **(H)** 5-hydroxymethylfurfural (5-HMF) contents of fermented black garlic extracts obtained using different probiotics and different fermentation times. Data are means ± SD (*n* = 3).

During fermentation, probiotics transform certain components to obtain energy, which can change the composition of the fermentation broth and thus influence the physicochemical and sensory properties. In this study, the number of viable bacteria; pH variation; and contents of total acid, amino-N, reducing sugars, total polyphenols, total flavonoids, and 5-HMF in the fermentation broth were investigated. The number of viable bacteria in the fermented black garlic extract first increased and then decreased with the extension of fermentation time and was highest on day 2 (Figure 3A). The viable counts of *L. plantarum* and the mixture of *L. plantarum* and *L. rhamnosus* reached ~10^15^ CFU/ml, whereas viable counts of *L. rhamnosus* reached 10^13^ CFU/ml, indicating that black garlic broth might be more suitable for survival of *L. plantarum* than *L. rhamnosus*. As shown in Figure 3B, the pH decreased significantly from 4.30 to 3.60 (*L. plantarum*), 3.93 (*L. rhamnosus*), and 3.60 (mixed *L. plantarum* and *L. rhamnosus*) after probiotic fermentation, possibly due to acids produced by *L. rhamnosus* and *L. plantarum* during fermentation. Therefore, the total acid content of the three fermentation broths was further evaluated. Figure 3C shows that the total acid content of the three fermentation broths increased with the extension of fermentation time, among which the total acid content of the bacteria fermentation mixture was the strongest, especially after day 1. After 4 days of fermentation, the total acid content in fermentation broths of *L. plantarum, L. rhamnosus*, and the mixture of *L. plantarum* and *L. rhamnosus* reached to 1.92 ± 0.01, 1.63 ± 0.01, and 2.04 ± 0.01 mg/ml, respectively. As shown in Figure 3D, when the black garlic extract was fermented by *L. rhamnosus* or the mixture of the two bacteria for 0–2 days, the amino-N content increased due to the destruction of protein structures. After 2 days, the amino-N content remained more or less stable. However, during fermentation by *L. plantarum*, the amino-N content first increased significantly to 8.69 ± 0.12 mg/ml from 0 to 1 day and then decreased linearly to 7.21 ± 0.12 mg/ml, possibly because the amino-N was further utilized by *L. plantarum*.

During the production of black garlic, the high temperature disrupts the glycosidic bonds of polysaccharides, which are converted into reducing sugars and oligosaccharides (25). As shown in Figure 3E, the reducing sugar content of the black garlic extract before fermentation was 109.05 ± 0.90 mg/ml. In the *L. plantarum* fermentation broth, the reducing sugar content decreased continuously. During *L. rhamnosus* fermentation, reducing sugars decreased sharply in the early stage (0–1 day) and then increased significantly with the extension of fermentation time. A possible reason is that the reducing sugars may be the preferred carbon source compared with sources such as polysaccharides. Therefore, the reducing sugars would be used first during the early fermentation stages until they are relatively low in abundance, at which point the probiotics may begin to consume other carbon sources (e.g., polysaccharides), which could produce high levels of reducing sugars (25, 32, 33).

Phenols and flavonoids are important plant components and, as free radical scavengers, provide significant benefits to human health (34). As shown in Figures 3F,G, in general, the total polyphenol and flavonoid contents increased significantly in the black garlic extract after fermentation by the three probiotics. The total polyphenol content was increased on day 1 of fermentation by *L. plantarum* or the mixture of the two bacteria and was decreased slightly thereafter. During fermentation by *L. rhamnosus*, there was a slight decrease in the total polyphenol content on day 1, possibly because the rate of free polyphenol and flavonoid production was lower than the oxidation rate of oxygen or other oxides in the air. Then, the polyphenol and flavonoid contents increased significantly to 2.69 ± 0.01 and 1.79 ± 0.08 mg/ml, respectively, which were markedly higher than those produced by fermentation by *L. plantarum* or the mixture of the two bacteria. These results were consistent with an earlier study (35). In plants, some polyphenols and flavonoids are usually combined or bound with polysaccharides, which are unable to be extracted. Probiotic fermentation would destroy the plant cell wall and release β-glucosidase or proteolytic enzyme, which may hydrolyze polyphenols and flavonoids into free and soluble forms, resulting in an increase in the overall contents of total polyphenols and total flavonoids (35–37).

The 5-HMF is a furan compound with aldehyde and hydroxymethyl functional groups formed mainly via the Maillard reaction and dehydration of sugars during the production of black garlic (9). In recent years, 5-HMF has been classified as a pollutant that may be carcinogenic. Moreover, 5-HMF has been reported to have effects such as liver and juvenile toxicity, DNA damage, and tumor transformation (38, 39). Therefore, the variation in 5-HMF content during fermentation was further investigated. As shown in Figure 3H, the 5-HMF content of black garlic extracts fermented by different probiotics for 4 days decreased significantly. The probiotic fermentation significantly reduced the 5-HMF content (*P* < 0.05). Compared with unfermented broth, the 5-HMF contents in the fermentation broth of *L. plantarum, L. rhamnosus*, and bacterial mixture were reduced by 37.66, 25.10, and 40.81%, respectively, demonstrating the more potent ability of *L. plantarum* to reduce 5-HMF compared with *L. rhamnosus*. Notably, for all physicochemical properties, the effects of fermentation by the bacterial mixture were similar to those of *L. plantarum* fermentation and differed significantly from those of *L. rhamnosus* fermentation. The main reason might be that black garlic was more suitable for the growth of *L. plantarum* than *L. rhamnosus*. Therefore, *L. plantarum* would gradually be the dominant strain, which played a major role in fermentation.

### Artificial and Electronic Sensory Evaluations of Fermented Black Garlic Broth

**Figure 4:**
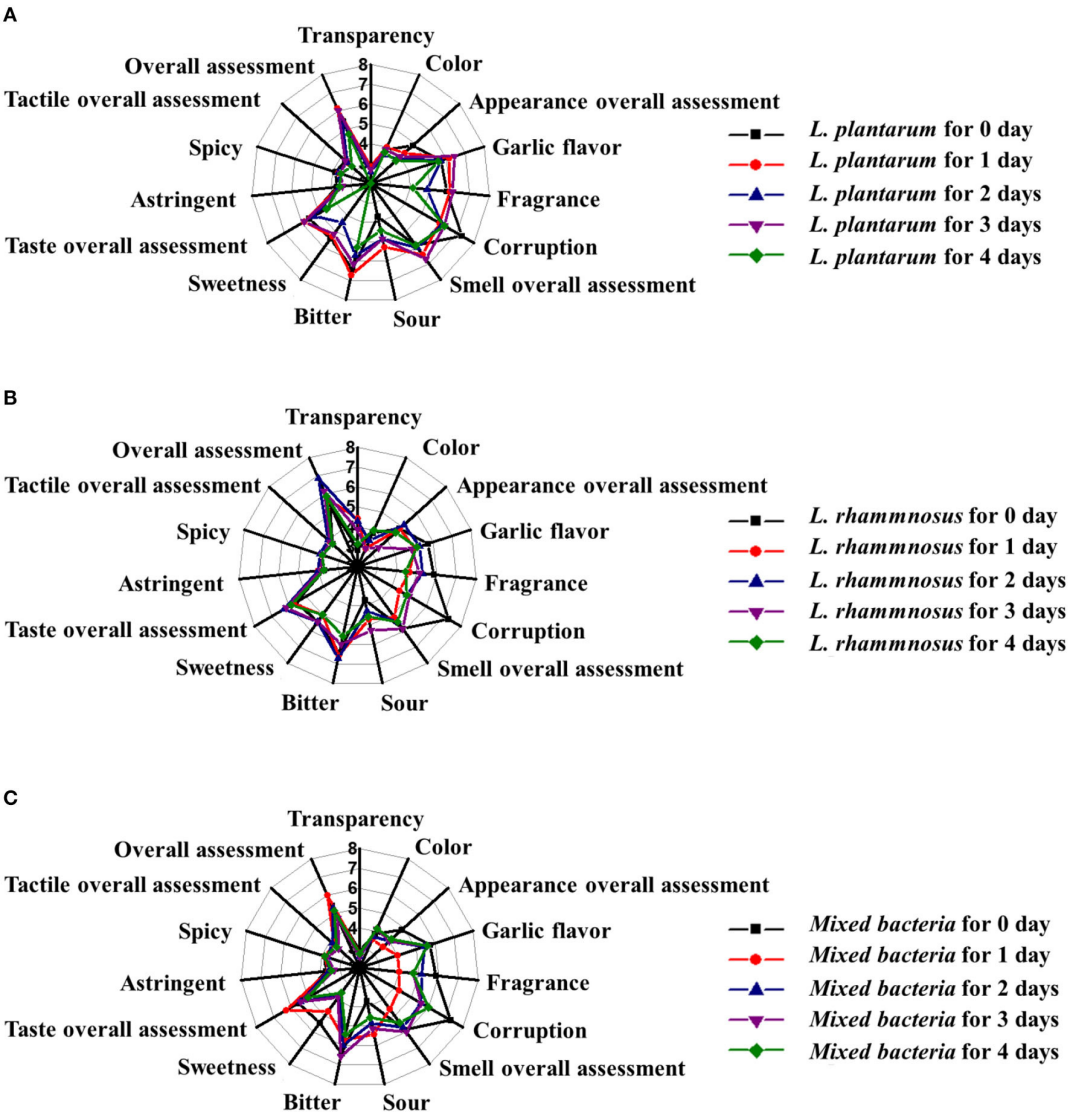
Artificial sensory evaluation of black garlic broths fermented by **(A)** *L. plantarum*, **(B)** *L. rhamnosus*, and **(C)** a mixture of the two bacteria at different times (0, 1, 2, 3, and 4 days). Data are means ± SD (*n* = 15).

Color, aroma, and taste are generally considered important characteristics that determine consumer evaluation of food (40). Artificial sensory evaluation is the most direct method for comprehensive analysis of food quality. Artificial sensory evaluation is based on the normal organs and processes of the human body, with advantages of simplicity, speed, economy, and practicality. Artificial sensory evaluation was conducted on the fermented black garlic broth extracts within 4 days of fermentation. The sensory characteristic scores are shown in Figure 4. The three fermentation products were sampled at 0, 1, 2, 3, and 4 days of fermentation. For fermentation by the same probiotic, the number of fermentation days did not largely affect the sweetness, spoilage, or sourness (*P* < 0.05). Compared with the unfermented black garlic extract (S1), the probiotic samples were slightly more acidic but were not considered unpleasant. The best overall sensory evaluations were obtained in fermentation broth samples from day 1 of fermentation by *L. plantarum* (S2), day 2 of fermentation by *L. rhamnosus* (S3), and day 1 of fermentation by co-cultured *Lactobacillus* (S4).

The artificial sensory evaluation is susceptible to human subjectivity, which results in significant differences and difficulty in reflecting the actual sensory characteristics. Electronic sensory evaluations (electronic eye, electronic nose, and electronic tongue) can simulate human body organs (eyes, nose, and tongue) in objective sensory evaluations of food, which are more convenient, effective, and accurate. To investigate the effects of probiotic fermentation on the sensory characteristics of black garlic, the differences between S1 and the three fermentation broths with the highest artificial sensory evaluation scores in appearance, flavor, and taste were characterized by the electronic eye, electronic nose, and electronic tongue, respectively.

**Figure 5:**
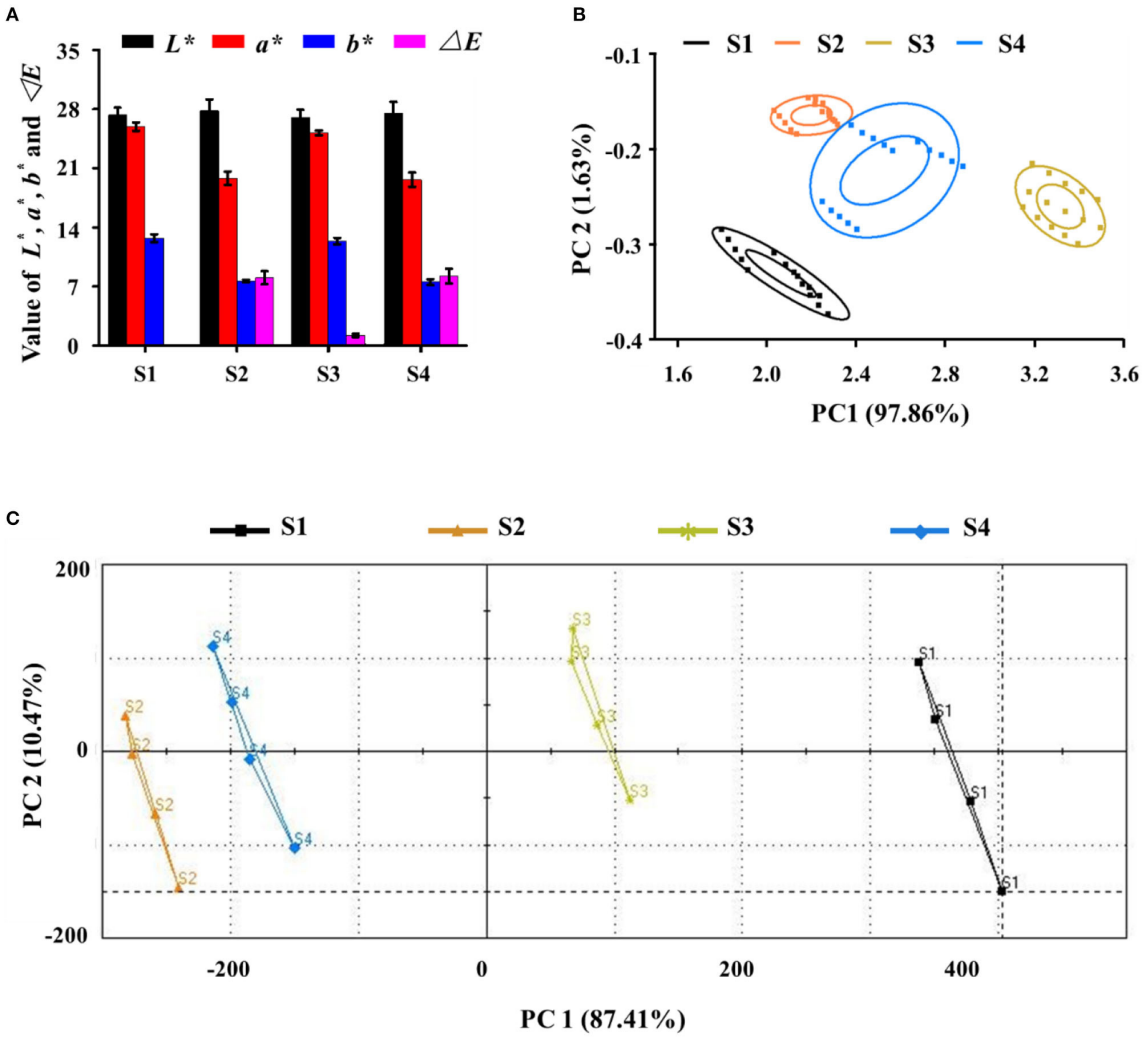
Electronic sensory evaluation of black garlic fermentation broths by **(A)** the electronic eye, **(B)** electronic nose (PCA), and **(C)** electronic tongue (PCA). Data are means ± SD (*n* = 4).

Food color has been shown to affect taste perception and is also one of the main determinants of food choice (41). The analysis results of the electronic eye are shown in Figure 5A. Compared with S1, the L^*^ values of S2, S3, and S4 did not change significantly, indicating that fermentation by the three probiotics did not significantly change the black color of the black garlic extract. The a^*^ and b^*^ values of S2 and S4 were decreased significantly (*P* < 0.05), whereas those of S3 were not decreased significantly, compared with S1. These indicated that fermentation by *L. plantarum* and a mixture of the two bacteria for 1 day significantly reduced the redness and yellowness of the fermentation broth, whereas fermentation by *L. rhamnosus* for 2 days did not change the fermentation broth color. The fermentation of *L. rhamnosus* had more effect on color appearance of black garlic broth. In fact, Δ*E* reflects the overall difference among the samples. Based on S1, the Δ*E* values of the three fermented samples had significant differences. These results demonstrate that the electronic eye can distinguish color changes among samples. Figures 5B,C shows the PCA results for the electronic nose and electronic tongue. In the two analyses, the cumulative contribution of the first two factors to the variance was above 99%, indicating significant differences among different samples. For the electronic nose analysis, the output of the 10 gas sensors showed that the response values of W5S, W1S, W1W, and W2W were critical for the four samples. The difference in aroma between unfermented and fermented products was derived mainly from nitrogen oxides, methane, sulfur compounds, and aromatic compounds. For the electronic tongue analysis, data from the seven sensors indicated that sourness, sweetness, and freshness were the key factors contributing to the PCA differences. The differences were related to the metabolites produced by probiotic fermentation. Therefore, the electronic eye, electronic nose, and electronic tongue were sufficient to distinguish samples of black garlic extract fermented by probiotics.

### Effects of Fermentation on Metabolites in Black Garlic Broth

**Figure 6:**
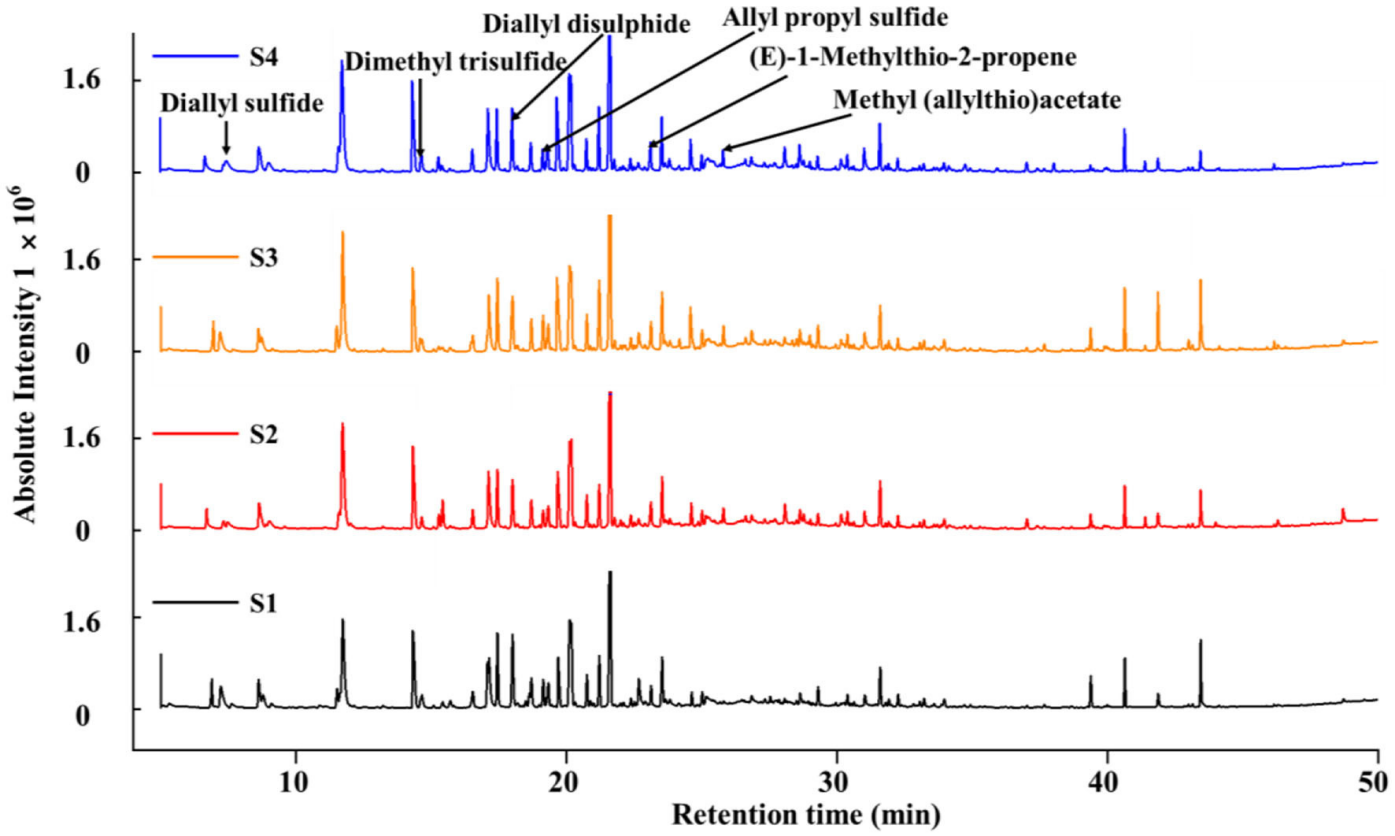
Gas chromatography–mass spectrometry (GC-MS) profiles of volatile compounds in black garlic fermentation broth.

The physicochemical properties and sensory characteristics of food are determined by the components therein. The variations of metabolites in S1, S2, S3, and S4 were further investigated by HS-SPME–GC-MS and LC–Q-TOF–MS/MS analyses. As a result, a total of 49 volatile metabolites were identified by HS-SPME–GC-MS and were divided into volatile sulfur compounds, flavor compounds, and three additional categories (Supplementary Table 1). The total sulfide content tended to decrease during fermentation, possibly due to the strong volatility. However, multiple sulfides could be detected in the four samples, and several with potent beneficial health effects, including diallyl sulfide, dimethyl trisulfide, 1-ethylthio-2-methyl-1-propene, 3-[(1-ethylthio) thio]-propanoic acid, 3-acetyl thio-2-methyl propanoic acid, and methyl allylthioacetate, were identified as the main sulfur compounds (Figure 6). Table 2 lists 24 flavor substances identified that may contribute to the flavor of the fermentation broth, and these substances were divided mainly into grass and flower, roast, fruit, acid, and other flavors. Among them, the roast flavor was the most prominent, mainly including furfural, 2-acetylfuran, and 5-methyl furfural, which accounted for ~3.80% of the relative total peak area. Both 2-acetylfuran and 5-methylfurfural are furfural derivatives, which are produced by the Maillard reaction. After fermentation, the contents of baking-flavored substances in S2 and S4 were decreased most significantly, indicating the potential effect of *L. plantarum*. The unfermented extract contained small amounts of green grass, floral, and fruity aroma substances. After the probiotics were fermented, they produced green grass, floral, and fruit aromas, such as benzaldehyde, phenethyl alcohol, and eugenol acetate, that improved the smell of the fermentation broth. Notably, the 5-HMF contents of the fermented samples (S2, S3, and S4) were 0.73, 1.24, and 0.26%, respectively (Table 2), which were lower than those of S1. This is consistent with the results shown in Figure 3H. *Lactobacillus* fermentation may be a new reliable method for reducing the content of 5-HMF.

**Table 2:** HS-SPME–GC-MS identification of the main functional volatile substances in black garlic broth.

**Compounds**	**RT (min)**	**CAS**	**Mass (Da)**	**Serial number**	**Formula**	**Retention index**	**Principal fragments**	**S1 (%)**	**S2 (%)**	**S3 (%)**	**S4 (%)**
**Green/floral flavor**											
2-Nonanone	15.290	821–55–6	142	7,667	C_9_H_18_O	1,052	43, 59, 99, 142	–	0.353	–	0.327
Nonanal	15.425	124–19–6	142	7,630	C_9_H_18_O	1,104	41, 70, 98, 124	–	0.738	–	–
Benzaldehyde	19.145	100–52–7	106	2,661	C_7_H_6_O	982	51, 77, 106	–	0.380	0.798	0.451
2-Undecanone	21.785	112–12–9	170	12,783	C_11_H_22_O	1,251	43, 58, 112, 127	–	0.157	–	0.191
Benzeneacetaldehyde	22.670	122–78–1	120	4,077	C_8_H_8_O	1,081	39, 65, 91, 120	0.968	0.149	0.381	–
Phenylethyl alcohol	30.160	1,960–12–8	122	5,604	C_8_H_10_O	1,136	39, 65, 91, 122	–	0.171	0.111	0.180
Eugenolacetate (6CI)	37.020	93–28–7	206	18,320	C_12_H_14_O_3_	1,552	43, 77, 91, 131	–	0.239	–	0.199
1-Dodecanol	37.670	112–53–8	186	15,467	C_12_H_26_O	1,457	55, 69, 97, 140	–	–	0.135	–
5-Hydroxymethylfurfural	43.445	67–47–0	126	6,369	C_6_H_6_O_3_	1,163	41, 69, 97, 126	1.242	0.727	1.237	0.260
**Fruity flavor**											
Limonene	8.785	138–86–3	136	6,614	C_10_H_16_	1,018	39, 68, 93, 136	0.633	–	0.478	–
Methyl 2-furoate	32.255	611–13–2	126	6,367	C_6_H_6_O_3_	909	39, 67, 95, 126	0.320	0.220	0.209	0.210
Farnesyl alcohol	41.395	4,602–84–0	222	59,386	C_15_H_26_O	1,710	41, 69, 93, 136	–	0.187	0.074	0.141
1-Tridecanol	41.870	112–70–9	200	17,566	C_13_H_28_O	1,556	56, 69, 97, 125	–	–	0.997	0.186
**Roasted flavor**											
Furfural	17.450	1,998–01–1	96	1,382	C_5_H_4_O_2_	831	38, 39, 67, 96	2.060	1.316	1.617	1.252
2-Acetylfuran	18.700	1,192–62–7	110	3,062	C_6_H_6_O_2_	878	39, 67, 95, 110	0.853	0.684	0.670	0.582
5-Methyl furfural	20.755	620–02–0	110	2,680	C_6_H_6_O_2_	920	53, 81, 109, 110	0.802	0.649	0.711	0.595
2-Acetyl pyrrole	31.595	1,072–83–9	109	2,603	C_6_H_7_NO	1,035	39, 66, 94, 109	0.145	0.087	0.138	0.091
**Sour flavor**											
Acetic acid	17.115	64–19–7	60	139	C_2_H_4_O_2_	576	43, 45, 60	1.729	1.905	1.610	1.900
Isovaleric acid	23.820	503–74–2	102	2,266	C_5_H_10_O_2_	811	43, 60, 87	–	–	–	0.219
Tetramethoxy ethane	27.535	1,069–12–1	148	14,322	C_6_H_124_	883	59, 105, 133, 148	0.139	–	–	–
**Other flavors**											
Cyclohexanone^*^ (earthy)	11.735	108–94–1	98	1,559	C_6_H_10_O	891	42, 55, 69, 98	4.750	4.750	4.750	4.750
2-Furanmethanol (bitter and spicy)	23.540	98–00–0	98	1,497	C_5_H_6_O_2_	885	41, 53, 81, 98	1.333	0.969	1.169	1.041
Hexanoic acid (sweaty)	28.625	142–62–1	116	3,619	C_6_H_12_O_2_	974	41, 60, 73, 88	0.294	0.339	0.357	0.515
1,4-Butanediol (bitterness)	30.630	110–63–4	90	1,176	C_4_H_10_O_2_	904	31, 42, 44, 71	–	–	0.067	–
6-Heptenoic acid (fatty)	31.015	1,119–60–4	128	7,106	C_7_H_12_O_2_	1,064	41, 68, 110, 128	0.298	0.345	0.239	0.515

* *Identification of compounds confirmed by analysis of standards. An en dash (–) indicates the compound was not detected*.

**Figure 7:**
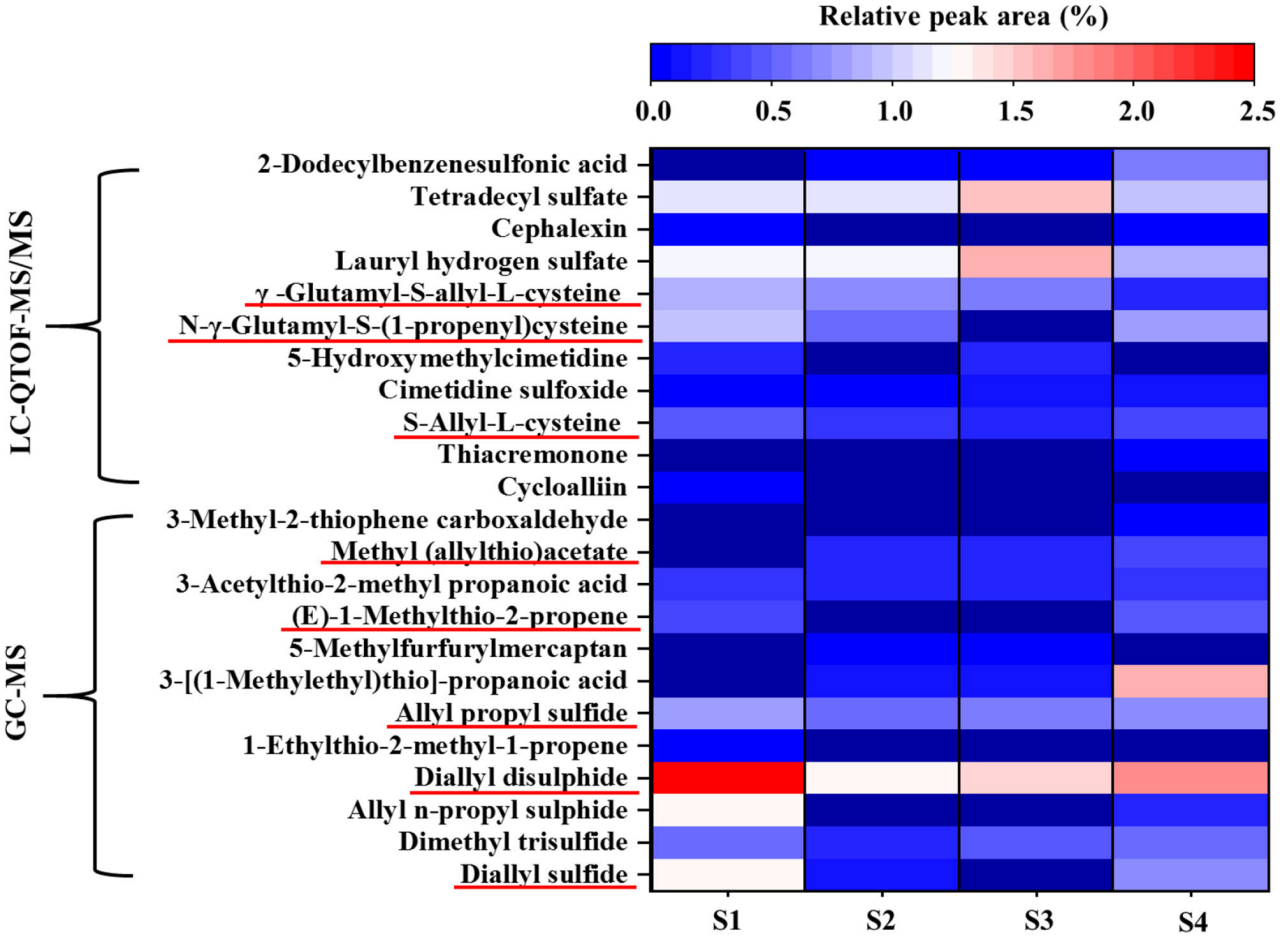
Heat map of relative peak area changes in the main sulfides of different black garlic broth extracts based on GC-MS and liquid chromatography–quadrupole time-of-flight–tandem mass spectrometry (LC–Q-TOF–MS/MS) analyses. The colors from blue to red indicate that the sulfide content gradually increased; dark blue indicates that sulfide was not detected.

Furthermore, 105, 94, 94, and 87 metabolites were identified by LC–QTOF–MS/MS in samples S1–S4, respectively, and were divided mainly into organic acids, amino acids, saccharides, organic sulfides, lipids, vitamins, etc. (Supplementary Table 2). *Lactobacillus* fermentation produced a large number of organic acids, which have potent antibacterial, anti-cholera, anti-inflammatory, hypoglycemic, antioxidant, and immune properties. Twenty-one organic acids were identified in the four samples (Table 3). The relative contents of several organic acids were enhanced in the three fermentation broths, including sinapoyl malate, isocitrate, lactic acid, cinnamic acid, creatine A, and 2,4-dimethyl-2-pentadecenoic acid. In particular, the newly produced lactic acid was relatively high in content in the three fermented samples: 14.13, 7.73, and 13.53% in samples S2, S3, and S4, respectively. The isocitrate content was increased dramatically in the probiotic fermentation broths, and this increased isocitrate content can enhance the tricarboxylic acid cycle and accelerate metabolic processes. Sinapoyl malate and cinnamic acid have also been shown to possess antioxidant and antimicrobial activities (42, 43). Notably, among the three fermentation broths, *L. plantarum* fermentation increased the organic acid content the most significantly. The total relative content of organic acids was as high as 60.60%, which is 1.7 times that in the unfermented broth. In comparison with the unfermented sample (S1), the relative content of 13,13-dimethyl-tetradecanoic acid, which exhibits a potent anti-cancer activity (44), was increased only in the *L. plantarum* fermentation broth, whereas it was almost undetectable in the other two fermentation samples. The total relative contents of amino acids and their derivatives were the highest in S2 (7.03%), followed by S3 (6.84%), S1 (3.36%), and S4 (2.65%), which was similar to the trend of the relative amino-N content. Peptides are amino acid chains smaller than proteins that play dominant roles in biological growth, development, reproduction, metabolism, and other life processes. Gly-Pro-Glu (GPE) has been identified as a neuroprotective peptide that can prevent glutamate from binding to the N-methyl-D-aspartate receptor. The relative contents of GPE in S2 (0.11%) and S3 (0.16%) were increased in comparison with S1 but were not detected in S4 (Table 3). Therefore, the fermentation of black garlic extracts by *L. plantarum* and *L. rhamnosus* may enhance their neuroprotective effects. Fermentation by *Lactobacillus* can also alter the composition of saccharides in black garlic broth. Notably, the relative content of sorbose was increased by 14.38-, 1.40-, and 26.6-fold in S2, S3, and S4, respectively, compared with S1; sorbose is an ideal sweetener for improving the taste of black garlic broth. Organic sulfides are the main functional ingredients involved in the health benefits of garlic. Here, N-γ-glutamyl-S-(1-propenyl) sulfide, γ-glutamyl-SAC, SAC, sulfoxide, and thiosulfate were identified as the main organic sulfides (Table 3). Figure 7 shows a heat map of the relative peak area changes of the main sulfides in the four samples. The relative contents of SAC in the S1, S2, S3, and S4 broths were 0.47, 0.34, 0.40, and 0.27%, respectively. SAC is a biologically active compound used in nutraceutical and medical applications for its antioxidant, anti-carcinogenic, and anti-hepatopathic activities (45). In addition, cycloalliin (0.07%) could not be detected after probiotic fermentation, which reduced the pungent odor. Sphingolipids are an important structural component of biofilms, as well as important functional molecules that participate in many signal transduction pathways and play important roles in programmed cell death and autophagy. Sphingosine is the simplest sphingolipid and a constituent of other sphingolipids together with fatty acids and the polar head group. The main sphingosines identified in the four samples (S1–S4) were phytosphingosine, C16 sphingosine, and sphingosine. The relative peak areas of phytosphingosine in samples S1–S4 were 6.32, 9.42, 8.55, and 9.55%, respectively, and a high phytosphingosine content helps maintain skin moisture and increases anti-inflammatory function. *L. rhamnosus* fermentation induced the most obvious increase in phytosphingosine content. The 3,4-dihydro-6-hydroxy-2,5,7,8-tetramethyl-2H-1-benzopyran-2-propanoic acid (α-CEHC) and γ-tocotrienol are important vitamins with antioxidant, anti-inflammatory, and reproductive functions. After probiotic fermentation, the α-CEHC content increased by at least twofold, with *L. plantarum* fermentation (S2) inducing a 2.72-fold increase, which may increase the antioxidant capacity of the probiotic fermentation broth. However, there was no obvious change in the content of γ-tocotrienol (Table 3). The results demonstrated that *Lactobacillus* could significantly influence the quality of black garlic by regulating the flavor and functional components. However, in actual consumption, various factors would affect the health benefits of food, including dietary habits, actual consumption (quantity/volume)/day of functional compounds, bioavailability, and metabolism *in vivo*). Therefore, the health effects of probiotic fermented black garlic broth *in vivo* need further investigation.

**Table 3:** Main substances identified in black garlic broth by LC–Q-TOF–MS/MS.

**Compounds**	**RT (min)**	**Formula**	***m*/*z***	**Mass (Da)**	**Adduct**	**S1 (%)**	**S2 (%)**	**S3 (%)**	**S4 (%)**
**Organic acids and derivatives**									
Sinapoyl malate	0.327	C_15_H_16_O_9_	363.068	340.079	[M + H]^+^	0.14	0.2	0.25	0.22
Isocitrate	0.368	C_6_H_8_O_7_	191.020	192.027	[M–H]^−^	13.98	25.04	15.31	14.91
L-Malic acid	0.397	C_4_H_6_O_5_	133.014	134.021	[M–H]^−^	1.35	–	–	–
Lactic acid	0.474	C_3_H_6_O_3_	89.024	90.032	[M–H]^−^	–	14.13	7.73	13.53
Cinnamic acid	0.713	C_9_H_8_O_2_	166.086	148.053	[M + H]^+^	0.06	0.14	0.19	0.15
Isohydrosorbic acid	0.566	C_6_H_10_O_2_	132.102	114.068	[M + H]^+^	1.17	0.98	1.67	1.27
Succinic acid	0.627	C_4_H_6_O_4_	117.019	118.027	[M–H]^−^	–	–	–	0.71
Cinnamic acid	0.713	C_9_H_8_O_2_	166.086	148.053	[M + H]^+^	0.06	0.14	0.19	0.15
**Amino acids and derivatives**									
Gly-Pro-Glu	0.353	C_12_H_19_N_3_O_6_	302.135	301.127	[M + H]^+^	0.08	0.11	0.16	–
Thr-Pro-Lys	7.380	C_15_H_28_N_4_O_5_	362.241	344.207	[M + NH_4_]^+^	–	–	–	0.22
Lys-Ile-Gln	8.245	C_17_H_33_N_5_O_5_	388.254	387.247	[M + H]^+^	–	–	–	0.15
Arg-Gln-Arg	9.254	C_17_H_34_N_10_O_5_	481.262	458.273	[M + H]^+^	0.22	0.2	–	0.16
Thr-Leu-Pro	13.150	C_15_H_27_N_3_O_5_	347.230	329.197	[M + NH_4_]^+^	–	–	–	0.2
Trp-Val-Trp	22.858	C_27_H_31_N_5_O_4_	507.272	489.238	[M + NH_4_]^+^	–	0.1	0.13	0.14
**Saccharides and derivatives**									
2-O-α-D-galactopyranuronosyl-L-rhamnose	0.315	C_12_H_22_O_11_	377.086	342.117	[M–H]^−^	0.52	0.39	0.24	1.04
Sorbose	0.362	C_6_H_12_O_6_	215.033	180.064	[M–H]^−^	0.07	1.01	0.10	1.86
3-Hydroxy-2H-pyran-2-one	0.370	C_5_H_4_O_3_	111.009	112.016	[M + H]^+^	0.79	0.9	0.95	–
α-L-arabinofuranosyl-(1-3)-β-D-xylopyranosyl-(1-4)-D-xylose	0.423	C_15_H_26_O_13_	413.130	414.137	[M–H]^−^	–	0.31	–	0.35
Tetrahydro-6-(2-hydroxy-16,19-dimethylhexacosyl)-4-methyl-2H-pyran-2-one	24.268	C_34_H_66_O_3_	540.535	522.501	M + NH_4_]^+^	–	0.29	–	0.47
**Organosulfur compounds**									
Cycloalliin	0.367	C_6_H_11_NO_3_S	178.053	177.046	[M + H]^+^	0.07	–	–	–
S-allyl-L-cysteine	0.425	C_6_H_11_NO_2_S	162.058	161.049	[M + H]^+^	0.47	0.34	0.40	0.27
N-γ-glutamyl-S-(1-propenyl) cysteine	1.962	C_11_H_18_N_2_O_5_S	289.087	290.094	[M – H]^−^	0.93	0.56	0.80	0.38
γ-Glutamyl-S-allyl-L-cysteine	2.077	C_11_H_18_N_2_O_5_S	291.101	290.094	[M + H]^+^	0.85	0.75	0.24	0.66
**Lipids and derivatives**									
C16 sphingosine	15.014	C16H35NO2	274.274	273.266	[M + H]^+^	0.99	1.07	10.48	11.66
Phytosphingosine	15.463	C18H39NO3	318.300	317.292	[M + H]^+^	6.32	9.42	8.55	9.55
C17 sphingosine	16.725	C_17_H_37_NO_2_	288.290	287.282	[M + H]^+^	0.13	–	–	–
Sphingosine	17.611	C_18_H_39_NO_2_	302.305	301.298	[M + H]^+^	0.44	0.48	1.59	0.55
**Vitamins and derivatives**									
α-CEHC	19.659	C_16_H_22_O_4_	301.141	278.151	[M + H]^+^	0.78	2.01	1.84	1.66
γ-Tocotrienol	19.853	C_28_H_42_O_2_	409.310	410.318	[M – H]^−^	1.48	1.49	1.49	1.46

*An en dash (–) indicates that the compound was not detected; α-CEHC, 3,4-dihydro-6-hydroxy-2,5,7,8-tetramethyl-2H-1-benzopyran-2-propanoic acid*.

## Conclusion

In this study, single-factor analysis and the response surface methodology were used to optimize the hot water extraction of soluble solids from black garlic. The optimal extraction conditions were found to be an extraction temperature of 99.96°C, a solid-to-liquid ratio of 1:4.38 g/ml, and an extraction time of 2.72 h. To obtain black garlic broth with more health-promoting effects, the effects of single-strain and mixed-strain fermentation by *L. plantarum* and *L. rhamnosus* on the physicochemical properties and flavor of black garlic extract broth were further investigated. The results showed that *L. plantarum* and *L. rhamnosus* reduced the pH of black garlic extract by increasing the total acid content, as well as increased the contents of amino-N, total polyphenols, and total flavonoids and reduced the 5-HMF content. Notably, for all physicochemical properties, the effects of fermentation by *L. plantarum* and *L. plantarum* plus *L. rhamnosus* were similar and differed significantly from the effects of fermentation by *L. rhamnosus*. Typically, fermentation by *L. plantarum* or mixed bacteria exhibited greater acid production and reduced 5-HMF content, while fermentation by *L. rhamnosus* resulted in higher total polyphenol and total flavonoid contents. Artificial sensory results demonstrated that the broths from S1, S2, and S4 received the best overall sensory evaluations. The contents of several components with unpleasant baking flavors such as furfural, 2-acetylfuran, and 5-methyl furfural were reduced, whereas those of components with green grass, floral, and fruit aromas were increased. More importantly, fermentation by *Lactobacillus* probiotics could significantly increase the contents of several functional components, including organic acids (e.g., lactic acid), amino acids (e.g., GPE), saccharides (e.g., sorbose), and vitamins (e.g., α-CEHC). *Lactobacillus* (*L. plantarum* and *L. plantarum*) could significantly influence the composition of flavor and functional components in black garlic extract. The health-promoting effects of black garlic may be consequently changed, which need a more comprehensive investigation, especially *in vivo* studies. This work will provide novel insights into the strategic design of new black garlic products and will facilitate the application of black garlic in functional foods.

## Data Availability Statement

The raw data supporting the conclusions of this article will be made available by the authors, without undue reservation.

## Author Contributions

LM made substantial contributions in the methodology, investigation, and writing of the original draft. CZ made substantial contributions in the validation, formal analysis, and writing of the manuscript. JC and JZ made substantial contributions in the conceptualization, supervision, writing, review, and editing of the manuscript, project administration, and funding acquisition. All authors contributed to the article and approved the submitted version.

## Conflict of Interest

The authors declare that the research was conducted in the absence of any commercial or financial relationships that could be construed as a potential conflict of interest.
