# Corrigendum: Effects of Anaerobic Fermentation on Black Garlic Extract by *Lactobacillus*: Changes in Flavor and Functional Components

**DOI:** 10.3389/fnut.2021.745272

**Published:** 2021-09-23

**Authors:** Li Ma, Chengying Zhao, Jifeng Chen, Jinkai Zheng

**Affiliations:** ^1^School of Life Sciences, Zhengzhou University, Zhengzhou, China; ^2^Institute of Food Science and Technology, Chinese Academy of Agricultural Sciences, Beijing, China

**Keywords:** black garlic extract, fermentation, *Lactobacillus*, sensory, functional components

In the published article, there was an error in the author's name Jinkai Zheng. Instead of “Jinkai Zheng,” it should be the corresponding author name “Jinkai Zheng^2*^”.

The authors apologize for this error and state that this does not change the scientific conclusions of the article in any way. The original article has been updated.

## Publisher's Note

All claims expressed in this article are solely those of the authors and do not necessarily represent those of their affiliated organizations, or those of the publisher, the editors and the reviewers. Any product that may be evaluated in this article, or claim that may be made by its manufacturer, is not guaranteed or endorsed by the publisher.

