# Supplementary material for: Effects of Anaerobic Fermentation on Black Garlic Extract by Lactobacillus: Changes in Flavor and Functional Components
Source: Front Nutr. 2021 May 21;8:645416. doi: 10.3389/fnut.2021.645416 (PMC8175794; doi:10.3389/fnut.2021.645416)
Supplement: Supplementary file 1 [file Data_Sheet_1.docx]

**Effects of anaerobic fermentation on black garlic extract by** ***Lactobacillus*: changes in flavour and functional components**

Li Ma^1, 2^, Chengying Zhao^2^, Jifeng Chen^1,*^, Jinkai Zheng^2,*^

^1^ School of Life Sciences, Zhengzhou University, Zhengzhou 450001, China

^2^ Institute of Food Science and Technology, Chinese Academy of Agricultural Sciences, Beijing 100193, China

Correspondence to: Jinkai Zheng; Jifeng Chen.

E-mail: jinkai8212@163.com (J. Zheng); chenjifeng@zzu.edu.cn (J. Chen).

**Supplementary Figures and Tables**

**Figure S1.** HPLC profiles of (A) 5-HMF standards and 5-HMF contents of the samples and (B) the standard linear relationship.

**Figure S2.** LC–Q-TOF–MS/MS profiles of the four black garlic exacts: the total ion current in (A) positive and (B) negative ion mode.

**Table S1.** HS-SPME–GC-MS identification of all volatile substances in black garlic broth.

**Table S2.** All substances identified in black garlic broth by LC–Q-TOF–MS/MS.

**
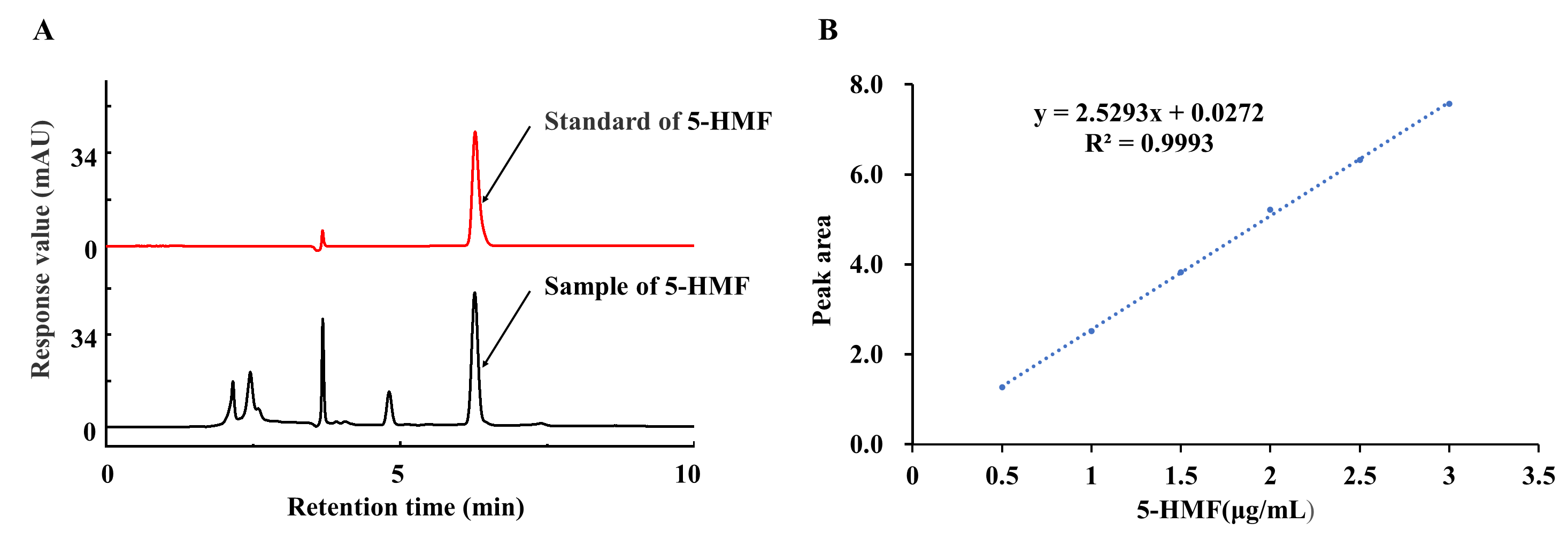
**

**Figure S1.** HPLC profiles of (A) 5-HMF standards and 5-HMF contents of the samples and (B) the standard linear relationship.


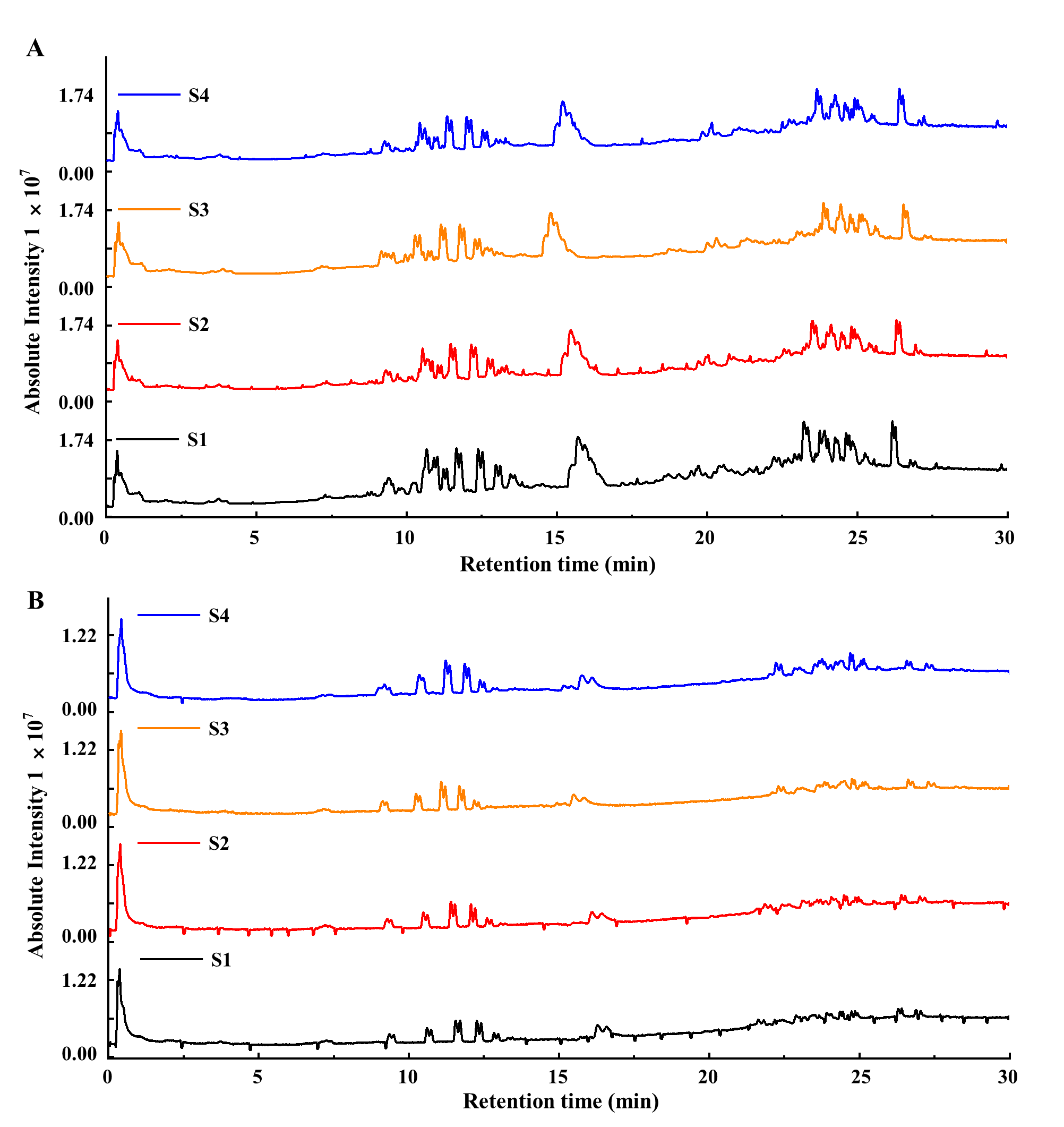


**Figure S2.** LC–Q-TOF–MS/MS profiles of the four black garlic exacts: the total ion current in (A) positive and (B) negative ion mode.

**Table S1.** HS-SPME–GC-MS identification of all volatile substances in black garlic broth.

| **Compounds** | **RT**  **(min)** | **CAS** | **Mass**  **(Da)** | **Serial number** | **Formula** | **Retention index** | **Principal fragments** | **S1**  **(%)** | **S2**  **(%)** | **S3**  **(%)** | **S4**  **(%)** |  |
| --- | --- | --- | --- | --- | --- | --- | --- | --- | --- | --- | --- | --- |
| **Derivatives for S-alk(en)yl -L-cysteine** | | | | | | | | | | | | |
| Diallyl sulfide | 7.310 | 592–88–1 | 114 | 3289 | C_6_H_10_S | 849 | 45, 73, 99, 114 | 1.262 | 0.157 | 0.765 | 0.105 |  |
| Dimethyl trisulfide | 14.665 | 3658–80–8 | 126 | 6269 | C_2_H_6_S_3_ | 972 | 45, 79, 111, 126 | 0.609 | 0.301 | 0.566 | 0.526 |  |
| Allyl 1-propyl sulphide | 17.080 | 27817–67–0 | 116 | 4606 | C_6_H_12_S | 859 | 41, 74, 116, 118 | 1.286 | – | 0.303 | – |  |
| Diallyl disulphide | 18.025 | 2179–57–9 | 146 | 13554 | C_6_H_10_S_2_ | 1099 | 41, 73, 113, 146 | 2.405 | 1.246 | 1.708 | 1.460 |  |
| 1-Ethylthio-2-methyl-1-propene | 18.500 | 27482–14–0 | 116 | 4624 | C_6_H_12_S | 854 | 45, 59, 87, 116 | 0.145 | – | – | – |  |
| Allyl propyl sulfide | 19.325 | 27817–67–0 | 146 | 13512 | C_6_H_12_S | 1026 | 41, 73, 87, 146 | 0.802 | 0.603 | 0.714 | 0.637 |  |
| 3-[(1-Methylethyl)thio]-propanoic acid | 20.180 | 24383–50–4 | 148 | 14299 | C_6_H_12_O_2_S | 1161 | 43, 75, 88, 133 | – | 0.191 | 1.556 | 0.191 |  |
| 5-Methylfurfurylmercaptan | 20.320 | 59303–05–8 | 128 | 7028 | C_6_H_8_OS | 1036 | 43, 65, 95, 127 | – | 0.106 | – | 0.125 |  |
| (E)-1-Methylthio-2-propene | 23.130 | 10152–76–8 | 88 | 998 | C_4_H_8_S | 678 | 39, 45, 73, 88 | 0.453 | – | 0.526 | – |  |
| 3-Acetylthio-2-methyl propanoic acid | 25.015 | 33325–40–5 | 162 | 11121 | C_6_H_10_O_3_S | 1297 | 43, 74, 102, 120 | 0.318 | 0.282 | 0.347 | 0.269 |  |
| Methyl (allylthio)acetate | 25.810 | 72867–23–3 | 146 | 13512 | C_6_H_10_O_2_S | 1026 | 45, 73, 114, 146 | – | 0.290 | 0.384 | 0.288 |  |
| 3-Methyl-2-thiophene carboxaldehyde | 27.335 | 5834–16–2 | 126 | 6355 | C_6_H_6_OS | 1072 | 45, 69, 97, 125 | – | – | 0.098 | – |  |
| **Flavor compounds** | | | | | | | | | | | | |
| **Green/floral flavor** | | | | | | | | | | | | |
| 2-Nonanone | 15.290 | 821–55–6 | 142 | 7667 | C_9_H_18_O | 1052 | 43, 59, 99, 142 | – | 0.353 | – | 0.327 |  |
| Nonanal | 15.425 | 124–19–6 | 142 | 7630 | C_9_H_18_O | 1104 | 41, 70, 98, 124 | – | 0.738 | – | – |  |
| Benzaldehyde | 19.145 | 100–52–7 | 106 | 2661 | C_7_H_6_O | 982 | 51, 77, 106 | – | 0.380 | 0.798 | 0.451 |  |
| 2-Undecanone | 21.785 | 112–12–9 | 170 | 12783 | C_11_H_22_O | 1251 | 43, 58, 112, 127 | – | 0.157 | – | 0.191 |  |
| Benzeneacetaldehyde | 22.670 | 122–78–1 | 120 | 4077 | C_8_H_8_O | 1081 | 39, 65, 91, 120 | 0.968 | 0.149 | 0.381 | – |  |
| Phenylethyl alcohol | 30.160 | 1960–12–8 | 122 | 5604 | C_8_H_10_O | 1136 | 39, 65, 91, 122 | – | 0.171 | 0.111 | 0.180 |  |
| Eugenolacetate (6CI) | 37.020 | 93–28–7 | 206 | 18320 | C_12_H_14_O_3_ | 1552 | 43, 77, 91, 131 | – | 0.239 | – | 0.199 |  |
| 1-Dodecanol | 37.670 | 112–53–8 | 186 | 15467 | C_12_H_26_O | 1457 | 55, 69, 97, 140 | – | – | 0.135 | – |  |
| 5-Hydroxymethylfurfural | 43.445 | 67–47–0 | 126 | 6369 | C_6_H_6_O_3_ | 1163 | 41, 69, 97, 126 | 1.242 | 0.727 | 1.237 | 0.260 |  |
| **Fruity flavor** | | | | | | | | | | | | |
| Limonene | 8.785 | 138–86–3 | 136 | 6614 | C_10_H_16_ | 1018 | 39, 68, 93, 136 | 0.633 | – | 0.478 | – |  |
| Methyl 2-furoate | 32.255 | 611–13–2 | 126 | 6367 | C_6_H_6_O_3_ | 909 | 39, 67, 95, 126 | 0.320 | 0.220 | 0.209 | 0.210 |  |
| Farnesyl alcohol | 41.395 | 4602–84–0 | 222 | 59386 | C_15_H_26_O | 1710 | 41, 69, 93, 136 | – | 0.187 | 0.074 | 0.141 |  |
| 1-Tridecanol | 41.870 | 112–70–9 | 200 | 17566 | C_13_H_28_O | 1556 | 56, 69, 97, 125 | – | – | 0.997 | 0.186 |  |
| **Roasted flavor** | | | | | | | | | | | | |
| Furfural | 17.450 | 1998–01–1 | 96 | 1382 | C_5_H_4_O_2_ | 831 | 38, 39, 67, 96 | 2.060 | 1.316 | 1.617 | 1.252 |  |
| 2-Acetylfuran | 18.700 | 1192–62–7 | 110 | 3062 | C_6_H_6_O_2_ | 878 | 39, 67, 95, 110 | 0.853 | 0.684 | 0.670 | 0.582 |  |
| 5-Methyl furfural | 20.755 | 620–02–0 | 110 | 2680 | C_6_H_6_O_2_ | 920 | 53, 81, 109, 110 | 0.802 | 0.649 | 0.711 | 0.595 |  |
| 2-Acetyl pyrrole | 31.595 | 1072–83–9 | 109 | 2603 | C_6_H_7_NO | 1035 | 39, 66, 94, 109 | 0.145 | 0.087 | 0.138 | 0.091 |  |
| **Sour flavor** | | | | | | | | | | | | |
| Acetic acid | 17.115 | 64–19–7 | 60 | 139 | C_2_H_4_O_2_ | 576 | 43, 45, 60 | 1.729 | 1.905 | 1.610 | 1.252 |  |
| Isovaleric acid | 23.820 | 503–74–2 | 102 | 2266 | C_5_H_10_O_2_ | 811 | 43, 60, 87 | – | – | – | 0.582 |  |
| Tetramethoxy ethene | 27.535 | 1069–12–1 | 148 | 14322 | C_6_H_12_O_4_ | 883 | 59, 105, 133, 148 | 0.139 | – | – | 0.595 |  |
| **Others flavor** | | | | | | | | | | | | |
| Cyclohexanone* (earthy) | 11.735 | 108–94–1 | 98 | 1559 | C_6_H_10_O | 891 | 42, 55, 69, 98 | 4.750 | 4.750 | 4.750 | 4.750 |  |
| 2-Furanmethanol (bitter and spicy) | 23.540 | 98–00–0 | 98 | 1497 | C_5_H_6_O_2_ | 885 | 441, 53, 81, 98 | 1.333 | 0.969 | 1.169 | 1.041 |  |
| Hexanoic acid (sweaty) | 28.625 | 142–62–1 | 116 | 3619 | C_6_H_12_O_2_ | 974 | 41, 60, 73, 88 | 0.294 | 0.339 | 0.357 | 0.515 |  |
| 1, 4-Butanediol (bitterness) | 30.630 | 110–63–4 | 90 | 1176 | C_4_H_10_O_2_ | 904 | 31，42，44, 71 | – | – | 0.067 | – |  |
| 6-Heptenoic acid (fatty) | 31.015 | 1119–60–4 | 128 | 7106 | C_7_H_12_O_2_ | 1064 | 41, 68, 110, 128 | 0.298 | 0.345 | 0.239 | 0.515 |  |
| **Others compounds** | | | | | | | | | | | | |
| 2-Propen-1-ol | 6.955 | 107–18–6 | 58 | 116 | C_3_H_6_O | 552 | 31, 39, 57, 59 | 0.606 | 0.339 | 0.583 | 0.363 |  |
| 1, 3-Bis(1, 1-dimethylethyl)benzene | 16.540 | 1014–60–4 | 190 | 15978 | C_14_H_22_ | 1334 | 41, 57, 91, 175 | 0.555 | 0.453 | 0.542 | 0.482 |  |
| Tetradecane | 22.005 | 629–59–4 | 198 | 17322 | C_14_H_30_ | 1413 | 57, 71, 99, 127 | – | 0.109 | – | – |  |
| 2(2-Ethoxyethoxy)ethanol | 22.380 | 111–90–0 | 134 | 6088 | C_6_H_14_O_3_ | 1012 | 45, 59, 104, 135 | 0.802 | 0.236 | – | 0.233 |  |
| (Z)-3-Decen-1-ol acetate | 28.080 | 81634–99–3 | 198 | 198 | C_12_H_22_O_2_ | 1389 | 43, 67, 96, 138 | – | 0.497 | – | 0.463 |  |
| 5-Ethyl-2-furaldehyde | 28.775 | 23074–10–4 | 124 | 5891 | C_7_H_8_O_2_ | 1020 | 39, 67, 109, 124 | – | 0.190 | – | 0.138 |  |
| Allyl methallyl ether | 31.915 | 14289–96–4 | 112 | 3526 | C_7_H_12_O | 750 | 41, 55, 69, 97 | 0.985 | 0.980 | 0.866 | 0.908 |  |
| Pyranone | 39.380 | 28564–83–2 | 144 | 12699 | C_6_H_8_O_4_ | 1269 | 43, 72, 101, 144 | 0.754 | 0.255 | 0.414 | 0.091 |  |
| 3, 5-Bis(1, 1-dimethylethyl)phenol | 40.640 | 1138–52–9 | 206 | 18381 | C_14_H_22_O | 1555 | 57, 77, 91, 135 | 1.079 | 0.725 | 1.071 | 0.667 |  |
| 2-(Dodecyloxy)ethanol | 43.005 | 4536–30–5 | 230 | 21146 | C_14_H_30_O_2_ | 1731 | 57, 71, 97, 140 | – | – | 0.182 | – |  |
| Dibutyl phthalate | 46.160 | 84–74–2 | 278 | 101150 | C_16_H_22_O_4_ | 2037 | 41, 76, 104, 149 | – | – | 0.111 | 0.075 |  |
| Hexadecanoic acid | 48.710 | 957–10–3 | 256 | 23305 | C_16_H_32_O_2_ | 1968 | 43, 73, 97, 129 | – | 0.358 | – | – |  |

“–” The compound was not detected. “*” Identification of compounds confirmed by analysis of standards.

**Table S2.** All substances identified in black garlic broth by LC–Q-TOF–MS/MS.

| **Compound** | **RT**  **(min)** | **Formula** | ***m/z*** | **Mass**  **（Da）** | **Adduct** | **S1**  **(%)** | **S2**  **(%)** | **S3**  **(%)** | **S4**  **(%)** |
| --- | --- | --- | --- | --- | --- | --- | --- | --- | --- |
| **Organic acids and derivatives** | | | |  |  |  |  |  |  |
| Erythronic acid | 0.305 | C_4_H_8_O_5_ | 135.030 | 136.037 | [M - H]^-^ | 1.16 | – | – | – |
| Sinapoyl malate | 0.327 | C_15_H_16_O_9_ | 363.068 | 340.079 | [M + H]^+^ | 0.14 | 0.20 | 0.25 | 0.22 |
| Isocitrate | 0.368 | C_6_H_8_O_7_ | 191.020 | 192.027 | [M - H]^-^ | 13.98 | 25.04 | 15.31 | 14.91 |
| L-Malic acid | 0.397 | C_4_H_6_O_5_ | 133.014 | 134.021 | [M - H]^-^ | 1.35 | – | – | – |
| Lactic acid | 0.474 | C_3_H_6_O_3_ | 89.024 | 90.032 | [M - H]^-^ | – | 14.13 | 7.73 | 13.53 |
| 4-Hydroxycinnamic acid | 0.536 | C_9_H_8_O_3_ | 182.082 | 164.048 | [M + H]^+^ | 0.08 | – | – | – |
| Isohydrosorbic acid | 0.566 | C_6_H_10_O_2_ | 132.102 | 114.068 | [M + H]^+^ | 1.17 | 0.98 | 1.67 | 1.27 |
| Succinic acid | 0.627 | C_4_H_6_O_4_ | 117.019 | 118.027 | [M - H]^-^ | – | – | – | 0.71 |
| Cinnamic acid | 0.713 | C_9_H_8_O_2_ | 166.086 | 148.053 | [M + H]^+^ | 0.06 | 0.14 | 0.19 | 0.15 |
| Crenatine A | 9.928 | C_34_H_40_N_4_O_4_ | 569.314 | 568.307 | [M + H]^+^ | – | 0.22 | 0.2 | 0.17 |
| 11-Hydroxy-dodecanoic acid | 10.366 | C_12_H_24_O_3_ | 234.206 | 216.173 | [M + H]^+^ | – | – | 0.09 | – |
| 13,13-Dimethyl-tetradecanoic acid | 15.349 | C_16_H_32_O_2_ | 274.273 | 256.240 | [M + H]^+^ | 15.75 | 17.78 | – | 0.16 |
| 4R-Methyl-hexadecanoic acid | 15.880 | C_17_H_34_O_2_ | 288.289 | 270.256 | [M + NH_4_]^+^ | 0.1 | – | – | – |
| 10-Oxo-14-methyl-pentadecanoic acid | 18.012 | C_16_H_30_O_3_ | 288.253 | 270.220 | [M + H]^+^ | – | 0.23 | – | – |
| 16-Hydroxy-10-oxohexadecanoic acid | 21.006 | C_16_H_30_O_4_ | 309.203 | 286.214 | [M + H]^+^ | 0.18 | 0.19 | 0.29 | 0.23 |
| 8-Nonynoic acid | 22.230 | C_9_H_14_O_2_ | 172.133 | 154.099 | [M + H]^+^ | 0.24 | – | – | – |
| Formic acid | 22.231 | CH_2_O_2_ | 44.998 | 46.006 | [M - H]^-^ | – | – | – | 0.33 |
| 2,4-Dimethyl-2-pentacosenoic acid | 23.744 | C_27_H_52_O_2_ | 431.384 | 408.395 | [M + H]^+^ | 0.08 | 0.18 | 0.17 | 0.14 |
| 3-Oxo-7α-hydroxy-5α-cholestan-26-oic acid | 24.255 | C_27_H_44_ O_4_ | 450.358 | 432.324 | [M + H]^+^ | 0.15 | 0.15 | 0.19 | 0.16 |
| 7β-Hydroxy-3-oxo-5beta-cholan-24-oic acid | 24.465 | C_24_H_38_O_4_ | 413.266 | 390.277 | [M + H]^+^ | 2.06 | 1.32 | 1.49 | 1.44 |
| Hexacosanedioic acid | 25.218 | C_26_H_50_O_4_ | 449.360 | 426.370 | [M + H]^+^ | 0.09 | – | – | – |
| **Amino acids and derivatives** |  |  |  |  |  |  |  |  |  |
| Asn Asn OH | 0.350 | C_13_H_14_N_4_O_8_ | 353.073 | 354.081 | [M - H]^-^ | – | 0.79 | 0.79 | – |
| Gly Pro Glu | 0.353 | C_12_H_19_N_3_O_6_ | 302.135 | 301.127 | [M + H]^+^ | 0.08 | 0.11 | 0.16 | – |
| 4-Guanidinobutanal | 0.389 | C_5_H_11_N_3_O | 130.167 | 129.160 | [M + H]^+^ | – | 0.28 | 0.39 | 0.34 |
| 4R-aminopentanoic acid | 0.390 | C_5_H_11_NO_2_ | 118.086 | 117.079 | [M + H]^+^ | 0.13 | 0.15 | 0.21 | – |
| 2,7-Anhydro-α-N-acetylneuraminic acid | 0.390 | C_11_H_17_NO_8_ | 290.088 | 291.095 | [M - H]^-^ | – | – | 0.09 | 0.32 |
| Pyrroline hydroxycarboxylic acid | 0.488 | C_5_H_7_NO_3_ | 130.050 | 129.042 | [M + H]^+^ | 0.53 | 4.37 | 4.27 | 0.78 |
| Hydroxyprolyl-tyrosine | 3.738 | C_14_H_18_N_2_O_5_ | 293.114 | 294.122 | [M - H]^-^ | 1.69 | 1.48 | 1.42 | – |
| Thr Pro Lys | 7.380 | C_15_H_28_N_4_O_5_ | 362.241 | 344.207 | [M + NH_4_]^+^ | – | – | – | 0.22 |
| Lys Ile Gln | 8.245 | C_17_H_33_N_5_O_5_ | 388.254 | 387.247 | [M + H]^+^ | – | – | – | 0.15 |
| Istamycin C1 | 8.641 | C_19_H_37_N_5_O_6_ | 432.280 | 431.273 | [M + H]^+^ | 0.24 | 0.21 | 0.17 | 0.19 |
| Arg Gln Arg | 9.254 | C_17_H_34_N_10_O_5_ | 481.262 | 458.273 | [M + H]^+^ | 0.22 | 0.20 | – | 0.16 |
| Thr Leu Pro | 13.150 | C_15_H_27_N_3_O_5_ | 347.230 | 329.197 | [M + NH_4_]^+^ | – | – | – | 0.20 |
| Cassine | 21.297 | C_18_H_35_NO_2_ | 320.256 | 297.267 | [M + NH_4_]^+^ | 0.32 | – | – | – |
| Trp Val Trp | 22.858 | C_27_H_31_N_5_O_4_ | 507.272 | 489.238 | [M + NH_4_]^+^ | – | 0.10 | 0.13 | 0.14 |
| Jubanine A | 23.174 | C_40_H_49_N_5_O_6_ | 696.379 | 695.372 | [M + H]^+^ | 0.07 | – | – | – |
| N-Oleoyl histidine | 23.217 | C_24_H_41_N_3_O_3_ | 458.279 | 419.316 | [M + H]^+^ | 0.08 | 0.13 | – | 0.15 |
| **Saccharides and derivatives** |  |  |  |  |  |  |  |  |  |
| 2-O-α-D-Galactopyranuronosyl-L-rhamnose | 0.315 | C_12_H_22_O_11_ | 377.086 | 342.117 | [M - H]^-^ | 0.52 | 0.39 | 0.24 | 1.04 |
| Sorbose | 0.362 | C_6_H_12_O_6_ | 215.033 | 180.064 | [M - H]^-^ | 0.07 | 1.01 | 0.10 | 1.86 |
| 3-Hydroxy-2H-pyran-2-one | 0.370 | C_5_H_4_O_3_ | 111.009 | 112.016 | [M + H]^+^ | 0.79 | 0.9 | 0.95 | – |
| 2-Hydroxy-propanedial | 0.375 | C_3_H_4_O_3_ | 87.009 | 88.016 | [M - H]^-^ | 0.5 | – | – | – |
| N-Acetylneuraminic acid | 0.384 | C_11_H_19_NO_9_ | 308.099 | 309.107 | [M - H]^-^ | 0.85 | 0.83 | – | – |
| Glycidaldehyde | 0.389 | C_3_H_4_O_2_ | 90.055 | 72.021 | [M + H]^+^ | – | – | 0.09 | – |
| 3-Hydroxy-N-methyl pyridinium glucuronide | 0.400 | C_12_H_17_NO_7_ | 305.134 | 287.100 | [M + H]^+^ | 0.30 | – | – | 0.46 |
| 2-Deoxymugineic acid | 0.421 | C_12_H_2_ON_2_O_7_ | 305.134 | 304.127 | [M + H]^+^ | – | – | 0.45 | – |
| α-L-Arabinofuranosyl-(1-3)-β-D-xylopyranosyl-(1-4)-D-xylose | 0.423 | C_15_H_26_O_13_ | 413.130 | 414.137 | [M - H]^-^ | – | 0.31 | – | 0.35 |
| Tetrahydro-6-(2-hydroxy-16,19-dimethylhexacosyl)-4-methyl-2H-pyran-2-one | 24.268 | C_34_H_66_O_3_ | 540.535 | 522.501 | [M + NH_4_]^+^ | – | 0.29 | – | 0.47 |
| **Organosulfur compounds** |  |  |  |  |  |  |  |  |  |
| Cycloalliin | 0.367 | C_6_H_11_NO_3_S | 178.053 | 177.046 | [M + H]^+^ | 0.07 | – | – | – |
| Thiacremonone | 0.411 | C_6_H_8_O_3_S | 178.053 | 160.019 | [M + NH_4_]^+^ | – | – | 0.13 | – |
| S-Allyl-L-cysteine | 0.425 | C_6_H_11_NO_2_S | 162.058 | 161.049 | [M + H]^+^ | 0.47 | 0.34 | 0.40 | 0.27 |
| Cimetidine sulfoxide | 0.470 | C_10_H_16_N_6_O_2_S | 307.096 | 284.107 | [M + H]^+^ | 0.11 | 0.12 | 0.15 | 0.15 |
| 5-Hydroxymethylcimetidine | 1.663 | C_10_H_16_N_6_OS | 291.101 | 268.112 | [M + H]^+^ | 0.27 | – | – | 0.25 |
| N-γ-Glutamyl-S-(1-propenyl) cysteine | 1.962 | C_11_H_18_N_2_O_5_S | 289.087 | 290.094 | [M - H]^-^ | 0.93 | 0.56 | 0.80 | 0.38 |
| γ -Glutamyl-S-allyl-L-cysteine | 2.077 | C_11_H_18_N_2_O_5_S | 291.101 | 290.094 | [M + H]^+^ | 0.85 | 0.75 | 0.24 | 0.66 |
| Lauryl hydrogen sulfate | 19.884 | C_12_H_26_O_4_S | 265.148 | 266.155 | [M - H]^-^ | 1.23 | 1.17 | 0.90 | 1.62 |
| Cephalexin | 21.439 | C_16_H_17_N_3_O_4_S | 191.581 | 347.095 | [M + H]^+^ | 0.12 | – | 0.12 | – |
| Tetradecyl sulfate | 22.012 | C_14_H_30_O_4_S | 293.179 | 294.187 | [M - H]^-^ | 1.1 | 1.12 | 0.93 | 1.48 |
| 2-Dodecylbenzenesulfonic acid | 22.96 | C_18_H_30_O_3_S | 325.184 | 326.191 | [M - H]^-^ | – | 0.11 | 3.62 | 0.12 |
| **Lipids and derivatives** |  |  |  |  |  |  |  |  |  |
| Glycerol | 2.340 | C_3_H_8_O_3_ | 115.037 | 92.048 | [M + H]^+^ | – | 0.04 | – | 0.03 |
| Octyl 2-methylpropanoate | 9.557 | C_12_H_24_O_2_ | 218.211 | 200.177 | [M + NH_4_]^+^ | 0.16 | – | – | – |
| Triethyl phosphate | 9.595 | C_6_H_15_O_4_P | 183.078 | 182.071 | [M + H]^+^ | – | 0.17 | 0.22 | – |
| PE-Cer(d14:2(4E,6E)/20:1(11Z)(2OH)) | 11.472 | C_36_H_69_N_2_O_7_P | 359.233 | 672.487 | [M - H]^-^ | 0.4 | 0.4 | 0.60 | 0.43 |
| PG(20:4(5Z,8Z,11Z,14Z)/18:3(9Z,12Z,15Z)) | 11.570 | C_44_H_73_O_10_P | 791.489 | 792.496 | [M - H]^-^ | 0.61 | 0.64 | 0.62 | 0.7 |
| PE(22:6(4Z,7Z,10Z,13Z,16Z,19Z)/22:2(13Z,16Z)) | 11.952 | C_49_H_82_NO_8_P | 844.588 | 843.580 | [M + H]^+^ | 0.09 | 0.11 | – | 0.11 |
| PI(22:4(7Z,10Z,13Z,16Z)/20:0) | 12.411 | C_51_H_91_O_13_P | 472.317 | 942.620 | [M - H]^-^ | 0.27 | 0.24 | 0.25 | 0.24 |
| Decyl isobutyrate | 12.687 | C_14_H_28_O_2_ | 246.243 | 228.209 | [M + NH_4_]^+^ | – | 0.19 | 1.37 | 0.24 |
| Dihydrojasmonic Acid, Methyl Ester | 12.908 | C_13_H_22_O_3_ | 225.150 | 226.157 | [M - H]^-^ | – | – | – | 0.38 |
| C16 Sphingosine | 15.014 | C_16_H_35_NO_2_ | 274.274 | 273.266 | [M + H]^+^ | 0.99 | 1.07 | 10.48 | 11.66 |
| Phytosphingosine | 15.463 | C_18_H_39_NO_3_ | 318.300 | 317.292 | [M + H]^+^ | 6.32 | 9.42 | 8.55 | 9.55 |
| MG(17:0/0:0/0:0)[rac] | 16.403 | C_20_H_40_O_4_ | 362.326 | 344.292 | [M + NH_4_]^+^ | 0.04 | – | – | – |
| C17 Sphinganine | 16.725 | C_17_H_37_NO_2_ | 288.290 | 287.282 | [M + H]^+^ | 0.13 | – | – | – |
| Estra-1,3,5(10)-triene-3,6beta,17beta-triol triacetate | 17.199 | C_24_H_30_O_6_ | 437.193 | 414.204 | [M + H]^+^ | 0.13 | – | – | – |
| Lauric diethanolamide | 17.57 | C_16_H_33_NO_3_ | 288.253 | 287.246 | [M + H]^+^ | 0.37 | 0.30 | 0.29 | 0.29 |
| Sphingosine | 17.611 | C_18_H_39_NO_2_ | 302.305 | 301.298 | [M + H]^+^ | 0.44 | 0.48 | 1.59 | 0.55 |
| Onchidal | 18.307 | C_17_H_24_O_3_ | 277.180 | 276.172 | [M + H]^+^ | 0.25 | 0.21 | 0.22 | 0.23 |
| (4E,8E,10E-d18:3) sphingosine | 20.799 | C_18_H_33_NO_2_ | 318.240 | 295.251 | [M + H]^+^ | – | – | – | 0.26 |
| D-erythro-Sphingosine C-15 | 20.855 | C_15_H_31_NO_2_ | 280.225 | 257.236 | [M + H]^+^ | 0.15 | – | – | – |
| N, N-dimethyl-safingol | 20.972 | C_20_H_43_NO_2_ | 330.336 | 329.329 | [M + H]^+^ | 0.48 | 0.44 | 0.55 | 0.41 |
| Tributyrin | 22.232 | C_15_H_26_O_6_ | 337.143 | 302.173 | [M - H]^-^ | – | – | – | 1.12 |
| Linoleamide | 22.596 | C_18_H_33_NO | 280.263 | 279.256 | [M + H]^+^ | 0.39 | 0.31 | 0.30 | 0.33 |
| Anandamide (20 : l, n-9) | 22.698 | C_22_H_43_NO_2_ | 376.319 | 353.329 | [M + H]^+^ | 0.12 | 0.11 | 0.15 | 0.13 |
| Palmitic amide | 22.919 | C_16_H_33_NO | 256.263 | 255.256 | [M + H]^+^ | 1.17 | 0.89 | 0.9 | 0.94 |
| Oleamide | 23.228 | C_18_H_35_NO | 282.279 | 281.272 | [M + H]^+^ | 3.94 | 3.85 | 4.02 | 4.39 |
| MG(0:0/16:0/0:0) | 23.280 | C_19_H_38_O_4_ | 353.266 | 330.277 | [M + H]^+^ | 0.08 | 0.07 | – | 0.10 |
| Dextromoramide | 23.286 | C_25_H_32_N_2_O_2_ | 415.237 | 392.248 | [M + H]^+^ | 0.10 | 0.09 | 0.12 | 0.14 |
| Methyl 2-pyrimidine carboxylate | 23.314 | C_6_H_6_N_2_O_2_ | 177.007 | 138.044 | [M + H]^+^ | 0.63 | 0.58 | – | 0.50 |
| 5alpha,17alpha-Pregn-2-en-20-yn-17-ol acetate | 23.328 | C_23_H_32_O_2_ | 339.232 | 340.239 | [M - H]^-^ | 0.88 | 0.75 | – | – |
| DG(15:0/20:1(11Z)/0:0) | 23.494 | C_38_H_72_O_5_ | 647.503 | 608.540 | [M + H]^+^ | 0.16 | 0.17 | 0.22 | 0.20 |
| Cer(d18:0/16:0(2OH)) | 23.657 | C_34_H_69_NO_4_ | 556.530 | 555.522 | [M + H]^+^ | 0.17 | 0.14 | 0.20 | 0.16 |
| Cer(d18:0/14:0) | 23.677 | C_32_H_65_NO_3_ | 534.486 | 511.496 | [M + H]^+^ | 0.06 | 0.53 | – | 0.51 |
| Stearamide | 23.832 | C_18_H_37_NO | 284.294 | 283.287 | [M + H]^+^ | 0.97 | 0.82 | 0.83 | 0.93 |
| Myxalamid B | 23.906 | C_25_H_39_NO_3_ | 424.282 | 401.293 | [M + H]^+^ | 0.11 | – | 0.11 | 0.12 |
| MG(0:0/16:0/0:0) | 24.038 | C_19_H_38_O_4_ | 353.266 | 330.277 | [M + H]^+^ | – | – | 0.07 | – |
| Cer(t20:0/16:0) | 24.084 | C_36_H_73_NO_4_ | 584.561 | 583.554 | [M + H]^+^ | 0.22 | 0.22 | 0.26 | 0.28 |
| Hexadecyl ferulate | 24.258 | C_26_H_42_O_4_ | 436.342 | 418.308 | [M + NH_4_]^+^ | 0.22 | 0.22 | 0.23 | 0.23 |
| Artemisyl propionate | 24.451 | C_13_H_22_O_2_ | 228.196 | 210.162 | [M + NH_4_]^+^ | – | – | 0.47 | – |
| Cer(d18:0/16:0) | 24.451 | C_34_H_69_NO_3_ | 540.534 | 539.527 | [M + H]^+^ |  | – | 0.80 | – |
| Cer(d18:0/18:0) | 24.488 | C_36_H_73_NO_3_ | 568.566 | 567.558 | [M + H]^+^ | – | 0.11 | – | – |
| DG(21:0/22:1(13Z)/0:0)[iso2] | 24.651 | C_46_H_88_O_5_ | 759.629 | 720.666 | [M + H]^+^ | 0.21 | 0.13 | 0.16 | 0.13 |
| 13Z-Docosenamide | 24.729 | C_22_H_43_NO | 338.341 | 337.334 | [M + H]^+^ | 3.27 | 3.27 | 3.24 | 3.35 |
| Cer(d20:0/18:0) | 24.741 | C_38_H_77_NO_3_ | 596.598 | 595.590 | [M + H]^+^ | – | 0.13 | – | 0.17 |
| TG(8:0/8:0/8:0) | 24.762 | C_27_H_50_O_6_ | 493.350 | 470.361 | [M + H]^+^ | 0.05 | – | – | – |
| PC(17:2(9Z,12Z)/22:4(7Z,10Z,13Z,16Z)) | 24.862 | C_47_H_83_NO_8_P | 821.596 | 820.589 | [M + H]^+^ | 0.1 | 0.09 | 0.12 | 0.10 |
| All trans decaprenyl diphosphate | 24.947 | C_50_H_90_O_7_P_2_ | 865.623 | 864.615 | [M + H]^+^ | 0.08 | 0.11 | 0.12 | 0.11 |
| Cabergoline | 25.168 | C_26_H_37_NO_2_ | 469.329 | 451.295 | [M + H]^+^ | 0.19 | 0.18 | – | – |
| **Vitamins and derivatives** |  |  |  |  |  |  |  |  |  |
| Dehydroascorbic acid | 0.375 | C_6_H_6_O_6_ | 173.010 | 174.017 | [M - H]^-^ | 0.62 | 0.37 | – | 0.61 |
| α-CEHC | 19.659 | C_16_H_22_O_4_ | 301.141 | 278.151 | [M + H]^+^ | 0.78 | 2.01 | 1.84 | 1.66 |
| γ-Tocotrienol | 19.853 | C_28_H_42_O_2_ | 409.310 | 410.318 | [M - H]^-^ | 1.48 | 1.49 | 1.49 | 1.46 |
| 1α,25-dihydroxy-2β-butoxyvitamin D3 /1α,25-dihydroxy-2β-butoxycholecalciferol | 25.254 | C_31_H_52_O_4_ | 506.420 | 488.386 | [M + NH_4_]^+^ | 0.17 | 0.14 | 0.25 | 0.21 |
| Theonellasterol G | 25.255 | C_30_H_50_O_4_ | 492.405 | 474.371 | [M + NH_4_]^+^ | 0.33 | 0.23 | 0.28 | 0.26 |
| 1α,25-dihydroxy-11α-(hydroxymethyl)vitamin D3 /  1α,25-dihydroxy-11α-(hydroxymethyl)cholecalciferol | 25.336 | C_28_H_46_O_4_ | 469.328 | 446.339 | [M + H]^+^ | 0.97 | 0.72 | 1.07 | 0.81 |
| **Nucleotides and derivatives** |  |  |  |  |  |  |  |  |  |
| 6-Hydroxyl-1,6-dihydropurine ribonucleoside | 0.413 | C_10_H_14_N_4_O_5_ | 269.088 | 270.095 | [M - H]^-^ | – | 2.43 | 1.47 | – |
| 5-Fluoro-5'-deoxyuridine | 8.796 | C_9_H_11F_N_2_O_5_ | 269.055 | 246.066 | [M + H]^+^ | 0.04 | 0.06 | – | 0.05 |
| 3,4,3',4'-Tetrahydrospirilloxanthin | 23.222 | C_42_H_64_O_2_ | 301.253 | 600.491 | [M + H]^+^ | 0.09 | 0.12 | – | 0.14 |
| **Alkaloids and derivatives** | | |  |  |  |  |  |  |  |
| 6-Gingerol | 14.924 | C_17_H_26_O_4_ | 293.176 | 294.183 | [M - H]^-^ | 1.14 | 1.23 | 1.38 | 2.02 |
| Jatrophatrione | 18.464 | C_20_H_26_O_3_ | 337.177 | 314.188 | [M + H]^+^ | 0.36 | 0.4 | 0.49 | 0.48 |
| Pipericine | 22.698 | C_22_H_41_NO | 336.326 | 335.319 | [M + H]^+^ | 0.08 | – | – | – |
| Nafoxidine | 24.181 | C_29_H_31_NO_2_ | 443.268 | 425.234 | [M + H]^+^ | 0.07 | – | – | – |
| **Antibiotics and derivatives** | | |  |  |  |  |  |  |  |
| 5-Hydroxymethyl-2-furaldehyde | 0.859 | C_6_H_6_O_3_ | 127.039 | 126.032 | [M + H]^+^ | – | 0.08 | – | – |
| SP 1802c | 10.423 | C_37_H_53_NO_3_ | 302.691 | 559.404 | [M + H]^+^ | 0.10 | – | 0.13 | – |
| Antanapeptin C | 11.150 | C_41_H_64_N_4_O_8_ | 763.464 | 740.474 | [M + H]^+^ | – | – | 0.14 | – |
| Foeniculoside VII | 22.394 | C_16_H_28_O_8_ | 347.171 | 348.178 | [M - H]^-^ | – | 1.38 | 1.52 | 3.43 |
| Stigmatellin Y | 22.566 | C_29_H_40_O_6_ | 507.271 | 484.282 | [M + H]^+^ | 0.32 | – | – | – |
| Dicrocin | 24.500 | C_32_H_44_O_14_ | 675.264 | 652.275 | [M + H]^+^ | – | – | 0.12 | – |
| **Others** |  |  |  |  |  |  |  |  |  |
| 1-[(ethenyloxy)-NNO-azoxy]-pyrrolidine | 0.284 | C_6_H_11_N_3_O_2_ | 175.118 | 157.085 | [M + NH_4_]^+^ | – | 0.8 | 1.06 | – |
| 3-Methyl-2-buten-1-ol | 0.302 | C_5_H_10_O | 104.107 | 86.073 | [M + H]^+^ | – | 1.05 | 1.30 | 1.23 |
| 3-(3,4-Dihydroxyphenyl)pyruvate | 0.339 | C_9_H_8_O_5_ | 219.026 | 196.037 | [M + H]^+^ | 0.16 | 0.16 | 0.36 | – |
| Pentane-2,4-dione | 0.395 | C_5_H_8_O_2_ | 118.086 | 100.052 | [M + NH_4_]^+^ | – | – | – | 0.17 |
| Gaboxadol | 0.487 | C_6_ H_8_ N_2_ O_2_ | 158.092 | 140.059 | [M + NH_4_]^+^ | 0.35 | 0.44 | 0.56 | 0.50 |
| Isoprene | 0.570 | C_5_H_8_ | 86.096 | 68.062 | [M + NH_4_]^+^ | 0.27 | 0.21 | 0.35 | 0.31 |
| 7Z-Tetradecen-1-ol | 14.999 | C_14_H_28_O | 230.248 | 212.214 | [M + H]^+^ | 0.69 | 0.81 | 0.98 | 1.15 |
| 7-Ethyltridecan-6-one | 15.675 | C_15_H_30_O | 244.264 | 226.230 | [M + NH_4_]^+^ | 0.08 | – | 0.12 | – |
| Aminopentol | 16.413 | C_22_H_47_NO_5_ | 406.352 | 405.345 | [M + H]^+^ | 0.27 | 0.21 | 0.23 | 0.23 |
| hexadeca-9-en-1-ol | 18.899 | C_16_H_32_O | 258.279 | 240.245 | [M + NH_4_]^+^ | 0.1 | – | – | – |
| 1-Nonadecene | 20.569 | C_19_H_38_ | 284.331 | 266.297 | [M + NH_4_]^+^ | 1.25 | 1.33 | – | 1.44 |
| 11-Octadecen-1-ol | 20.697 | C_18_H_36_O | 286.311 | 268.277 | [M + NH_4_]^+^ | 0.14 | – | 0.16 | – |
| Furmecyclox | 20.771 | C_14_H_21_NO_3_ | 250.145 | 251.152 | [M - H]^-^ | – | – | – | 0.41 |
| Imidiocarb | 21.437 | C_19_H_20_N_6_O | 387.133 | 348.170 | [M + H]^+^ | 0.11 | – | – | – |
| 10Z-Heneicosene | 21.633 | C_21_H_42_ | 312.362 | 294.329 | [M + NH_4_]^+^ | 0.1 | – | – | – |
| Gabapentin | 21.984 | C_9_H_17_NO_2_ | 172.133 | 171.126 | [M + H]^+^ | 0.14 | 0.24 | 0.22 | 0.26 |
| 7-Pentacosene | 23.133 | C_25_H_50_ | 368.425 | 350.391 | [M + NH_4_]^+^ | 0.19 | 0.20 | 0.47 | 0.25 |
| (-)-Jamine | 23.831 | C_21_H_35_N_3_ | 368.247 | 329.284 | [M + NH_4_]^+^ | 0.09 | 0.09 | 0.11 | – |
| 6S,7R-Epoxy-3Z,9Z-octadecadiene | 24.013 | C_18_H_32_O | 282.279 | 264.245 | [M + NH_4_]^+^ | 0.09 | 0.19 | – | 0.08 |
| N-Hexadecanoylpyrrolidine | 24.121 | C_20_H_39_NO | 310.311 | 309.303 | [M + H]^+^ | 0.11 | – | – | – |

“–” The compound was not detected.

“α-CEHC” that is 3,4-dihydro-6-hydroxy-2,5,7,8-tetramethyl-2H-1-benzopyran-2-propanoic acid;

“γ-Tocotrienol” that is (2R)-3,4-dihydro-2,7,8-trimethyl-2-[(3E,7E)-4,8,12-trimethyl-3,7,11-tridecatrien-1-yl]-2H-1-benzopyran-6-ol.
